# Hypertension Increases Susceptibility to Lead-Induced Microglial Polarization via ANT1-Mediated Mitochondrial DNA/cGAS/STING Signaling

**DOI:** 10.34133/research.1026

**Published:** 2025-12-15

**Authors:** Yuwei Zhao, Fan Shi, Han Hao, Zeming Wang, Jianan Li, Bingrui Liu, Yong Niu, Weixuan Wang, Yanshu Zhang

**Affiliations:** ^1^ School of Public Health, North China University of Science and Technology, Tangshan, Hebei 063210, P.R. China.; ^2^ National Institutions for Occupational Health and Poison Control, China CDC, Beijing 100050, P.R. China.; ^3^ Hebei Key Laboratory of Occupational Health and Safety for Coal Industry, North China University of Science and Technology, Tangshan, Hebei 063210, P.R. China.; ^4^ School of Life Sciences, North China University of Science and Technology, Tangshan, Hebei 063210, P.R. China.

## Abstract

Hypertensive individuals are concurrently at risk of heavy metal exposure, particularly lead (Pb). However, it remains unclear whether individuals with hypertension are more sensitive to the neurotoxicity induced by Pb exposure. Here, we established a hypertension model of mice with Pb exposure for 2, 4, 8, 12, and 24 weeks to detect the temporal and spatial changes in microglial polarization. Pb-exposed and hypertensive mice exhibited a notably higher proportion of M1 as early as 4 and 8 weeks, but a lower proportion of M2 as early as 4 and 12 weeks. Notably, hypertension exacerbated Pb-induced microglial polarization, which reached a plateau at 12 weeks. Elevated inducible nitric oxide synthase and reduced arginase-1 protein expressions were observed in the prefrontal cortex, hippocampus, and hypothalamus of the Pb, hypertension (HTN), and Pb+HTN groups, with the most pronounced alterations occurring in the prefrontal cortex. Furthermore, treatment with minocycline (microglial polarization inhibitor) markedly alleviated the depression-like behavior of mice in the Pb+HTN group. Bioinformatics analysis revealed that the differentially expressed genes of microglia following Pb and angiotensin II combined exposure were mainly enriched in the cyclic GMP-AMP synthase/stimulator of interferon genes (cGAS/STING) pathway. In addition, the protein expression of the cGAS/STING pathway was significantly upregulated both in vitro and in vivo, especially in the Pb+HTN group. Inhibition of cGAS significantly alleviated the increase in M1-type microglia and the decrease in M2-type microglia in hypertensive mice with Pb exposure. Meanwhile, mitochondrial DNA (mtDNA) leakage of microglia was observed under Pb and angiotensin II exposure, and the activation of the cGAS/STING pathway by mtDNA was proven to play an important role in microglial polarization. Adenine nucleotide translocase 1 (ANT1) was identified as the pivotal protein regulating mtDNA leakage via bioinformatics analyses and was proven by in vitro and in vivo studies. Collectively, hypertensive mice are more sensitive to Pb-induced neurotoxicity, which is mediated by the ANT1-regulated mtDNA/cGAS/STING signaling pathway, which then subsequently induces microglial polarization.

## Introduction

Hypertension ranks among the most widespread chronic conditions, affecting nearly 40% of the adult population globally [1]. Evidence shows that hypertension, a key risk factor for neurodegenerative disorders, promotes oxidative stress and subsequent neuroinflammatory responses, ultimately driving neurobehavioral impairment [2,3]. Hypertensive individuals are notably more susceptible to environmental pollutants [4,5], particularly heavy metals. Among them, Pb exposure has been most prominently associated with elevated blood pressure [6]. Numerous epidemiological studies have demonstrated a significant correlation between chronic Pb exposure and increased arterial blood pressure [7]. Neuroinflammation plays a critical role in hypertension-related neurobehavioral impairment [8]. Although numerous studies have characterized neuroinflammatory alterations in the brain following either Pb exposure or hypertension alone, the neuroinflammatory response and underlying mechanisms in hypertensive individuals following Pb exposure remain unexplored.

As the primary innate immune defenders within the brain, microglia are involved in mediating inflammation in the central nervous system [9,10]. Microglia are highly heterogeneous and can polarize into different phenotypes, including pro-inflammatory M1 microglia and anti-inflammatory M2 microglia [11–14]. A study demonstrated that the M1 microglial polarization of hypertensive mice resulted in nerve injury [15]. Pb exposure has been shown to increase inducible nitric oxide synthase (iNOS; M1 marker) expression while decreasing arginase-1 (Arg1; M2 marker) expression [16–18]. The above findings suggest that microglial polarization may be involved in the neurobehavioral impairment of hypertensive individuals with Pb exposure. Notably, current research on microglial polarization and neuroinflammation has predominantly focused on changes at a single time point or within a specific brain region, often overlooking the spatiotemporal dynamics of microglial polarization. Therefore, systematically elucidating the spatiotemporal patterns of microglial polarization in hypertensive mice with Pb exposure is of great significance for developing precise prevention and intervention strategies for populations at risk.

The cyclic GMP-AMP synthase/stimulator of interferon genes (cGAS/STING) signaling cascade represents a conserved mechanism for inflammatory defense throughout evolution [19]. Inhibition of the cGAS/STING pathway has been shown to regulate microglial polarization and alleviate brain injury following subarachnoid hemorrhage [20]. In addition, blocking the cGAS/STING pathway also reduces M1 microglial polarization, thereby attenuating neuroinflammation in the neuropathic pain model of mice [21]. However, the role of the cGAS/STING signaling pathway in mediating microglial polarization in hypertensive mice with Pb exposure remains largely unknown and warrants further investigation.

cGAS is a cytosolic DNA sensor that is activated upon recognition of cytosolic DNA. Mitochondrial DNA (mtDNA) represents the sole extranuclear genetic material, consisting of a circular double-stranded structure [22–24]. Unlike nuclear DNA, mtDNA exhibits greater instability and susceptibility to oxidative damage [25,26]. An increased mtDNA copy number has been observed in individuals occupationally exposed to Pb compared to that in unexposed controls [27]. Additionally, circulating levels of mitochondrial genes such as CytB and ND6 were significantly elevated in the plasma of spontaneously hypertensive rats [28]. However, it remains unclear whether the cytoplasmic mtDNA levels of microglia increase in hypertensive mice with Pb exposure.

mtDNA leakage is closely associated with the excessive opening of the mitochondrial permeability transition pore (mPTP) and abnormal mitochondrial fusion and fission [29–31]. Studies have shown that microplastics could disrupt mitochondrial membrane potential (MMP) and excessive opening of the mPTP, leading to mtDNA release into the cytoplasm of skin cancer cells. Environmental stressors trigger the overactivation of mPTP channels, resulting in mtDNA extrusion via BCL2 [32]. Down-regulation of MFN2 in microglia disrupts mitochondrial fusion and fission balance, leading to the release of mtDNA leakage after spinal cord injury [33]. However, the key proteins mediating mtDNA leakage in the microglia of hypertensive mice with Pb exposure remain to be identified.

Herein, the present study establishes hypertensive mice with Pb exposure to explore the spatiotemporal changes in microglial polarization. Transcriptome sequencing and bioinformatics analysis were used to identify the key mechanisms of microglial polarization in hypertensive mice with Pb exposure. The findings will provide a basis for protective targeting of nerve damage in hypertensive individuals following Pb exposure.

## Results

### Hypertensive mice are more susceptible to Pb-induced microglial polarization in a time-dependent manner

**Fig. 1.**
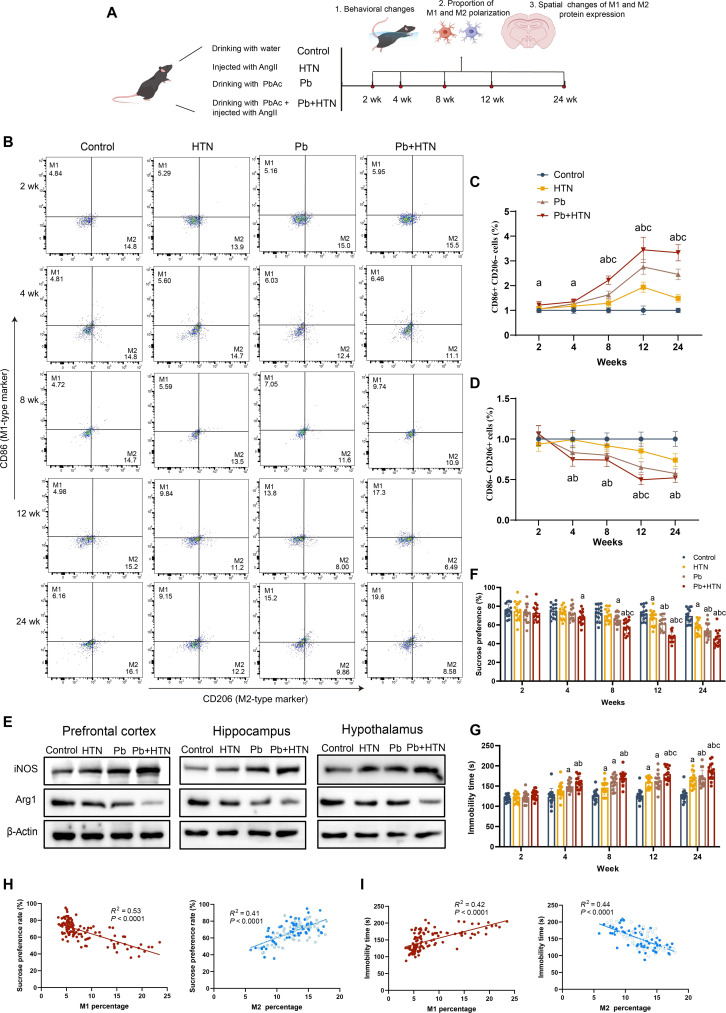
The polarization of microglia in the brain of hypertensive mice with Pb exposure. (A) Timeline of the experiment. (B) M1 and M2 microglia in mice from different experimental groups by flow cytometry analysis. (C) The diagrams display the percentages of M1 and (D) M2 microglia in different groups (*n* = 6). (E) The protein expression of inducible nitric oxide synthase (iNOS) and arginase-1 (Arg1) in the prefrontal cortex (PFC), hippocampus, and hypothalamus (*n* = 3). (F) Sucrose preference rate. (G) Immobility time in the forced swimming test (FST) in different groups (*n* = 15). (H and I) The correlation analysis between the percentages of M1 and M2 and neurobehavior. ^a^*P* < 0.05 vs control group; ^b^*P* < 0.05 vs hypertension (HTN) group; ^c^*P* < 0.05 vs Pb group. AngII, angiotensin II; PbAc, lead acetate.

To clarify the atlas of M1 and M2 microglia of hypertensive mice with and without Pb exposure for 2, 4, 8, 12, and 24 weeks, we examined the expression of M1 and M2 phenotypes in brain tissues (Fig. 1A). Hypertensive mice maintained a systolic blood pressure (SBP) ≥140 mmHg during the entire exposure duration (Fig. S1B). As a result, Pb-exposed mice or hypertensive mice exhibited a notably higher proportion of M1 microglia as early as 4 or 8 weeks. Hypertensive mice with Pb exposure exhibited an increase in M1 microglia as early as 2 weeks and peaked at 12 weeks. Meanwhile, a reduced trend in M2 microglia was seen in Pb-exposed mice or hypertensive mice from 4 or 12 weeks, respectively. Furthermore, hypertension could exacerbate the decline of M2 microglia in Pb-exposed mice at 4 weeks and keep a reduction until 24 weeks (Fig. 1B to D, Fig. S1A, and Tables S1 and S2). These data highlight that Pb exposure induced a more pronounced microglial polarization in hypertensive mice, showing a time-dependent pattern that plateaued at 12 weeks.

We quantified the expression of iNOS (an M1 microglial marker) and Arg1 (an M2 microglial marker) across multiple murine brain regions, specifically the prefrontal cortex (PFC), hippocampus, and hypothalamus, in different groups. Comparative analysis revealed a significant upregulation of iNOS coupled with the down-regulation of Arg1 in these brain areas among hypertension (HTN) and Pb-exposed groups relative to those in control animals. The protein expression of iNOS was significantly increased, while Arg1 expression was reduced in the PFC, hippocampus, and hypothalamus of mice in both the HTN and Pb groups. In addition, iNOS expression in hypertensive mice with Pb exposure was higher than that in hypertensive and Pb-exposed mice, especially in the PFC. Arg1 expression in the PFC and hypothalamus in hypertensive mice with Pb exposure was significantly lower than that in the Pb or the HTN group (Fig. 1E and Fig. S1C and D). These data indicate that the PFC might be the most sensitive brain region in terms of microglial polarization.

Given that inflammatory cytokine production is linked to microglial polarization, we examined the expression of interleukin-1β (IL-1β) and interleukin-6 (IL-6) in different brain tissues. Results showed that the expression of IL-1β and IL-6 was upregulated in various brain regions, with the most pronounced elevation observed in the PFC of hypertensive mice with Pb exposure (Fig. S1E to G). Next, we further assessed the temporal changes in depression-like behaviors in mice. The results showed that hypertension progressively reduced sucrose preference and increased immobility time in the forced swimming test (FST) among Pb-exposed mice, with initial changes detectable at 4 weeks and the most pronounced effects observed at 12 weeks (Fig. 1F and G). The data exhibited precise spatiotemporal synchronization with microglial polarization, particularly in sensitive brain regions (PFC). Additionally, correlation analysis demonstrated a significant association between M1/M2 microglial polarization and neurobehavioral alterations (Fig. 1H and I).

### Inhibition of microglial polarization alleviates the depression-like behaviors in hypertensive mice with Pb exposure

**Fig. 2.**
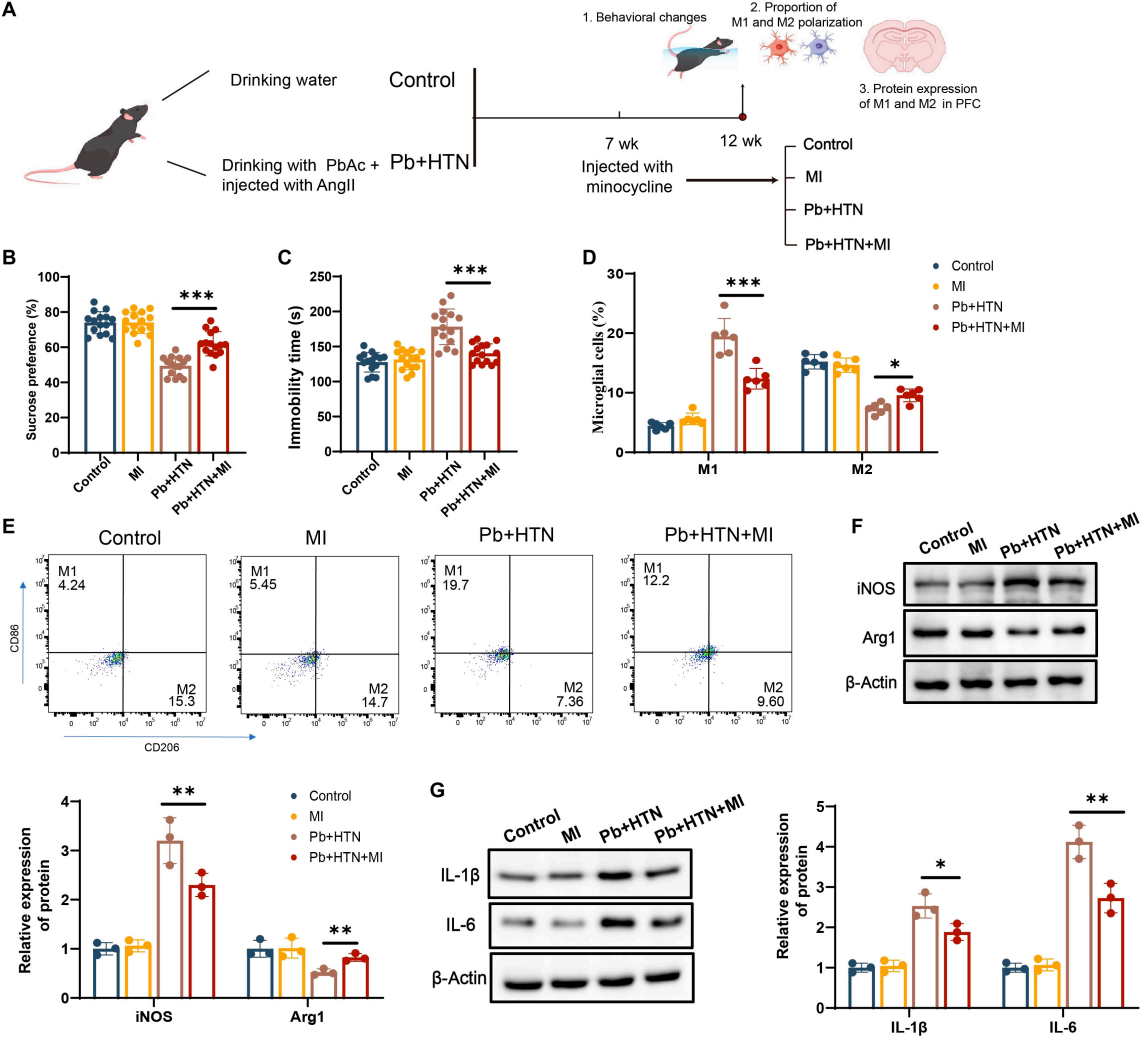
Effect of minocycline on microglial polarization in hypertensive mice with Pb exposure. (A) Timeline of the experiment. (B) Sucrose preference rate (*n* = 15). (C) Immobility time in FST (*n* = 15). (D and E) M1 and M2 microglia in mice from different experimental groups by flow cytometry analysis (*n* = 6). (F and G) The protein expression of iNOS, Arg1, interleukin-1β (IL-1β), and interleukin-6 (IL-6) in the PFC (*n* = 3). *P < 0.05 vs indicated group; **P < 0.01 vs indicated group; ***P < 0.001 vs indicated group. MI, minocycline.

To elucidate the role of microglial polarization in the nerve injury of hypertensive mice with Pb exposure, experimental mice were injected with minocycline (microglial polarization inhibitor [34]) to inhibit microglial polarization (Fig. 2A). Behavioral results showed that minocycline treatment could decrease the immobility time in FST and increase the sucrose preference rate in hypertensive mice with Pb exposure (Fig. 2B and C). In addition, minocycline treatment decreased M1 microglia and rescued M2 microglia in hypertensive mice with Pb exposure (Fig. 2D and E). Consistently, minocycline also resulted in a lower protein expression of iNOS and a higher Arg1 protein expression in the PFC (Fig. 2F). Meanwhile, the protein expression of IL-1β and IL-6 was also attenuated in the PFC of hypertensive mice with Pb exposure (Fig. 2G). Subsequently, BV2 cells were treated with lead acetate (PbAc) or angiotensin II (AngII) at different concentrations. The results indicated that exposure to either Pb or AngII reduced the survival rate of BV2 cells in a concentration-dependent fashion (Fig. S2A and B). Finally, a dose of 10 μmol/l Pb and 100 nmol/l AngII was selected for subsequent cell experiments. The expression of iNOS and pro-inflammatory cytokines was significantly upregulated, while Arg1 expression was markedly down-regulated in BV2 cells following co-exposure to Pb and AngII (Fig. S2C to E). Minocycline significantly inhibited these phenomena (Fig. S2F to H).

These results suggest that minocycline intervention suppresses microglial polarization induced by Pb exposure and hypertension and alleviates depressive-like behaviors in hypertensive mice with Pb exposure, indicating the possible role of microglial polarization in Pb- and hypertension-induced neurotoxicity.

### cGAS/STING was identified as the critical pathway of microglial polarization following Pb and AngII exposure

**Fig. 3.**
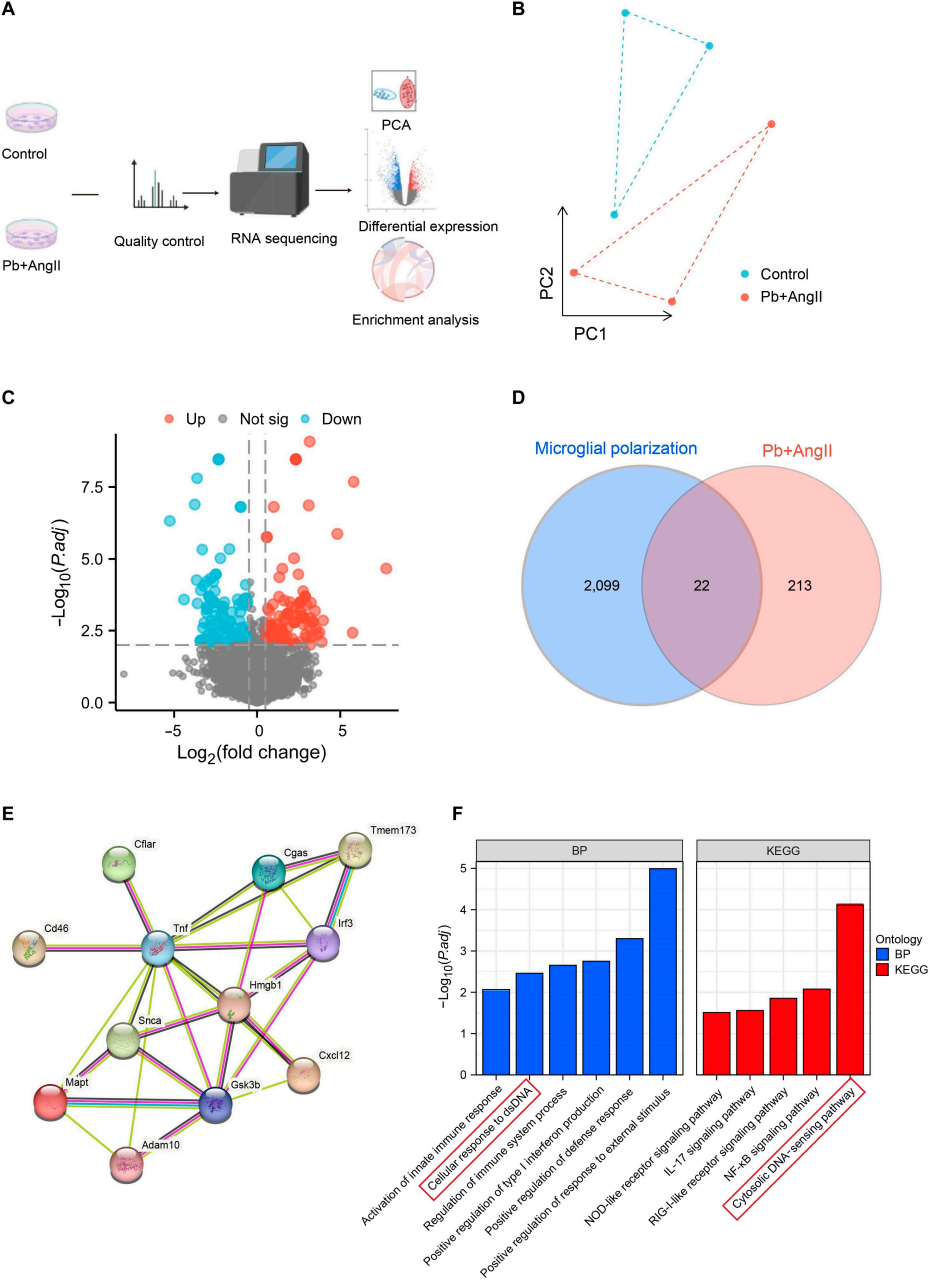
The cyclic GMP-AMP synthase/stimulator of interferon genes (cGAS/STING) pathway was identified as the most important pathway of microglial polarization following Pb and AngII co-exposure. (A) Diagram depicting the RNA sequencing (RNA-seq) workflow. (B) Principal component analysis results derived from microglial RNA-seq data. (C) Volcano plot showing differentially expressed genes (DEGs) in microglia following treatment with Pb+AngII. (D) Venn diagram showing overlaps among microglial-polarization-related DEGs identified in the Pb+AngII group. (E) A molecular interaction network depicting relationships among key molecules. (F) The enrichment analysis of biological processes (BP) and Kyoto Encyclopedia of Genes and Genomes (KEGG). PCA, principal component analysis; dsDNA, double-stranded DNA; IL-17, interleukin-17; RIG-I-like, retinoic acid-inducible gene-I-like; NF-κB, nuclear factor-κB.

**Fig. 4.**
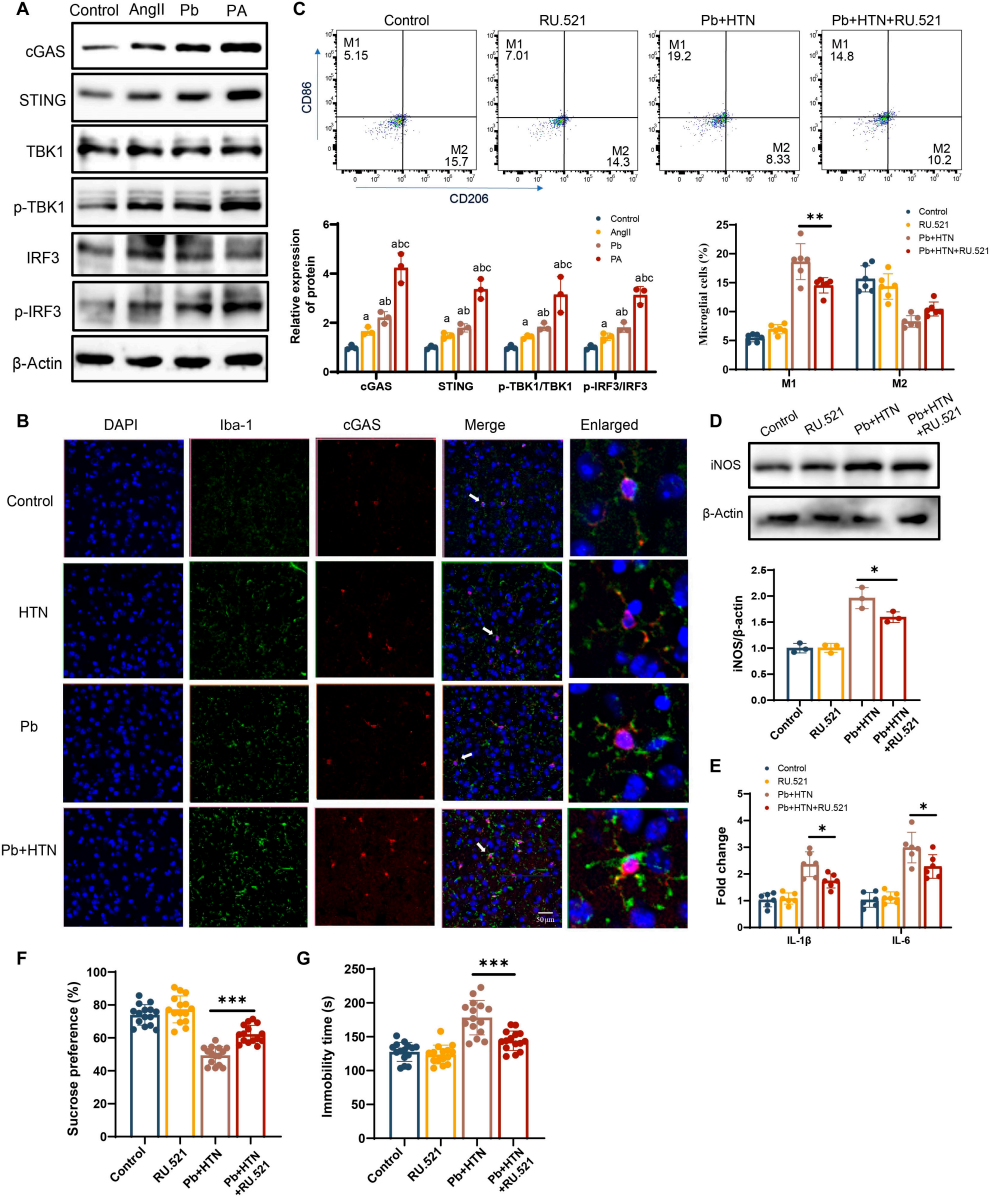
Pb and AngII can induce microglial polarization via the cGAS/STING pathway. (A) cGAS, STING, TANK-binding kinase 1 (TBK1) (both phosphorylated and total forms), and interferon regulatory factor 3 (IRF3) (phosphorylated and total) protein expression in Pb- or AngII-treated microglial cells by western blot (*n* = 3). PA, Pb+AngII. (B) cGAS and ionized calcium-binding adapter molecule 1 (Iba-1) co-localization analysis via immunofluorescence in the PFC. (C) M1 and M2 microglia in mice from different experimental groups by flow cytometry analysis (*n* = 6). (D) Western blot showing iNOS expression in the PFC of each group’s mice (*n* = 3). (E) The messenger RNA (mRNA) levels of IL-1β and IL-6 were measured by quantitative polymerase chain reaction (qPCR) (*n* = 6). (F) Sucrose preference rate (*n* = 15). (G) Immobility time in FST (*n* = 15). ^a^*P* < 0.05 vs control group; ^b^*P* < 0.05 vs HTN group; ^c^*P* < 0.05 vs Pb group; *P < 0.05 vs indicated group; **P < 0.01 vs indicated group; ***P < 0.01 vs indicated group. DAPI, 4′,6-diamidino-2-phenylindole.

To investigate the regulatory mechanisms governing microglial polarization in response to Pb and hypertension exposure, we employed transcriptome sequencing to characterize key regulatory pathways (Fig. 3A). The results showed good repeatability among the biological replicates (Fig. 3B). We performed differential analysis strategy to identify differentially expressed genes (DEGs) between the control and Pb+AngII groups, which identified 235 DEGs, including 116 upregulated and 119 down-regulated genes (Fig. 3C). To identify genes implicated in microglial polarization, the DEGs were matched to microglial-polarization-related genes, ultimately pinpointing 22 candidate genes (Fig. 3D and E). Moreover, DEG-related microglial polarization was primarily enriched in the cytoplasmic DNA-sensing pathway (Fig. 3F). Given the pivotal role of cGAS/STING in cytoplasmic DNA sensing, we examined the protein expression of the cGAS/STING pathway. The protein expression of cGAS, STING, phospho-TANK-binding kinase 1 (p-TBK1), and phospho-interferon regulatory factor 3 (p-IRF3) was elevated in the AngII and Pb groups compared with that in the control group. Notably, the expression of cGAS, STING, p-TBK1, and p-IRF3 in microglia co-exposed to Pb and AngII was significantly higher than that of the AngII and Pb groups. Among them, the cGAS protein expression in the Pb+AngII group was increased to 2.56-fold and 1.92-fold of those of the AngII and Pb groups, respectively (Fig. 4A). We also observed a marked increase in cGAS fluorescence intensity in PFC microglia from hypertensive mice with Pb exposure (Fig. 4B).

To further elucidate the role of the cGAS/STING pathway in regulating microglial polarization, RU.521 (a cGAS inhibitor) was administered to hypertensive mice with Pb exposure. Inhibition of cGAS reduced microglial polarization and decreased IL-1β and IL-6 levels in hypertensive mice with Pb exposure compared to those in the untreated Pb+HTN group (Fig. 4C to E). Meanwhile, inhibition of cGAS significantly increased sucrose preference and decreased FST immobility time in hypertensive mice with Pb exposure (Fig. 4F and G). Subsequently, BV2 cells with cGAS knocked down were established and they were treated with Pb or AngII alone or co-exposed for 24 h (Fig. S3A and B). The results demonstrated that cGAS knockdown significantly suppressed the upregulation of iNOS, IL-1β, and IL-6 in microglia co-exposed to Pb and AngII (Fig. S3C and D). The above findings underscore that cGAS/STING plays a vital role in the microglial polarization of hypertensive mice with Pb exposure, which promoted neuroinflammation.

### mtDNA leakage activates cGAS/STING signaling in microglial polarization following AngII and Pb exposure

**Fig. 5.**
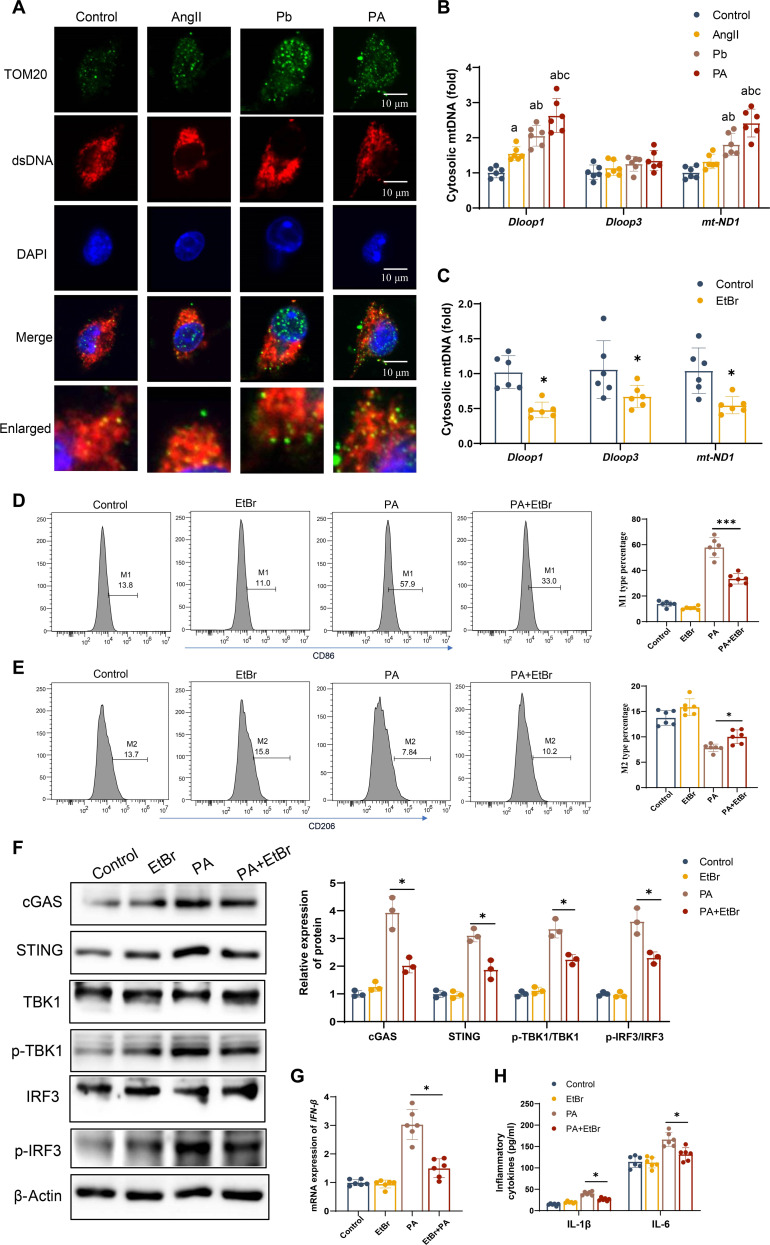
Co-exposure to AngII and Pb triggered mitochondrial DNA (mtDNA) leakage, activating the cGAS/STING signaling pathway in microglia. (A) Immunofluorescence staining for dsDNA/translocase of outer mitochondrial membrane 20 (TOM20) in BV2 cells. (B) Copy number of cytoplasmic mtDNA in microglia. (C) The mRNA levels of *Dloop1*, *Dloop3*, and *mt-ND1*. (D and E) Flow cytometry analysis of M1 microglia and M2 microglia in BV2 cells (*n* = 6). (F) The protein expression of cGAS, STING, p-TBK1, and p-IRF3 in microglia (*n* = 3). (G) The mRNA levels of *IFN-β* (*n* = 6). (H) Inflammatory factors in the supernatant of BV2 cells (*n* = 6). ^a^*P* < 0.05 vs control group; ^b^*P* < 0.05 vs AngII group; ^c^*P* < 0.05 vs Pb group; *P < 0.05 vs indicated group; ***P < 0.01 vs indicated group. EtBr, ethidium bromide.

It was reported that cytoplasmic mtDNA was one of the initial factors in terms of activating the cGAS/STING pathway [35]. To investigate whether mtDNA leaks from the mitochondria of microglia, BV2 cells were exposed to AngII, Pb, or their combination. We found that the accumulation of cytosolic double-stranded DNA in BV2 cells from the AngII, Pb, and Pb+AngII groups was significantly more than that in the control group (Fig. 5A). In addition, cytosolic mtDNA levels were significantly elevated in both Pb- and AngII-exposed groups, and this effect was further amplified in the Pb+AngII group (Fig. 5B). Moreover, microglia exhibited significant MMP loss, adenosine triphosphate (ATP) depletion, mitochondrial superoxide (mtSOX) elevation, and opening of the mPTP upon Pb or AngII exposure, with aggravated effects in the co-exposure group (Figs. S4A to C and S5A).

To determine whether mtDNA leakage plays a critical role in activating the cGAS/STING pathway in BV2 cells following combined treatment with Pb and AngII, we used ethidium bromide (EtBr) to eliminate mtDNA. This chemical agent selectively prevents mtDNA replication in microglia [36]. The mtDNA expression was inhibited by approximately 50% in microglia treated with EtBr (0.2 μg/ml) for 48 h (Fig. 5C). The data revealed that the proportion of M1-type microglia decreased and that of M2-type microglia increased following the addition of EtBr in microglia co-exposed to Pb and AngII (Fig. 5D and E). Moreover, the protein expression of cGAS, STING, p-TBK1, and p-IRF3 was lower in BV2 cells with Pb and AngII co-exposure after EtBr treatment (Fig. 5F). The elevated inflammatory factors (IL-6 and IL-1β) in BV2 cells induced by Pb and AngII were also inhibited (Fig. 5H). These results indicate that co-exposure to AngII and Pb resulted in the release of mtDNA into the cytosol, thereby activating the cGAS/STING pathway and leading to microglial polarization.

### ANT1 plays a critical role in the mtDNA leakage of microglia following Pb and AngII co-exposure

**Fig. 6.**
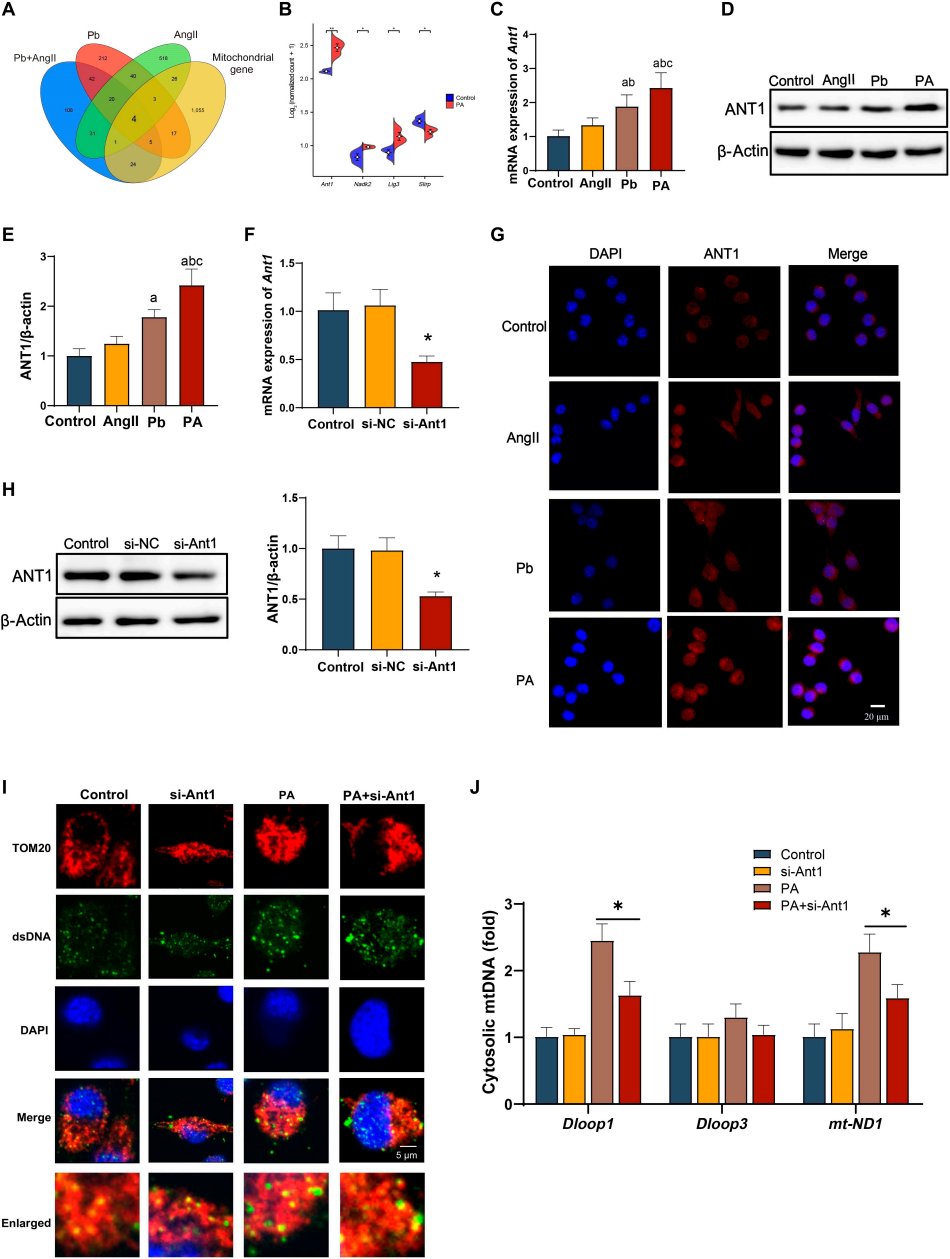
The role of adenine nucleotide translocase 1 (ANT1) in the leakage of mtDNA from BV2 cells induced by Pb and AngII co-exposure. (A and B) Polarized microglia regulate mitochondrial DEGs. (C) The mRNA levels of *Ant1* (*n* = 6). (D and E) Western blot (WB) showing the expression of ANT1 in BV2 cells (*n* = 3). (F) After knocking down *Ant1* in BV2 cells, their mRNA levels and (H) protein expression (*n* = 6 or 3). si-NC, nontargeting control small interfering RNA. (G) Immunofluorescent labeling of ANT1 and nuclear counterstaining with DAPI in BV2 microglial (*n* = 3). (I) Immunofluorescence staining for dsDNA/TOM20 in BV2 cells (*n* = 3). (J) Copy number of cytoplasmic mtDNA in microglia (*n* = 6). ^a^*P* < 0.05 vs control group; ^b^*P* < 0.05 vs AngII group; ^c^*P* < 0.05 vs Pb group; *P < 0.05 vs indicated group; **P < 0.01 vs indicated group.

To screen genes regulating the release of mtDNA into the cytosol of microglia following Pb and AngII co-exposure, RNA sequencing (RNA-seq) of microglia treated with Pb, AngII, and Pb+AngII co-exposure was conducted. As a result, *Ant1*, *Nadk2*, *Lig3*, and *Slirp* were potentially identified in terms of mtDNA leakage and the gene expression of *Ant1* showed the greatest change compared with other genes in microglia following Pb and AngII co-exposure (Fig. 6A and B). Further, BV2 cells treated with Pb and AngII alone or co-exposure showed that both the protein and messenger RNA levels of *Ant1* were elevated in the Pb and Pb+AngII groups, particularly in the co-exposure group (Fig. 6C to E). This suggests that adenine nucleotide translocase 1 (ANT1) may be a critical gene involved in the release of mtDNA into the cytosol of microglia under these conditions. Next, we knocked down *Ant1* expression in BV2 cells, achieving a reduction of 48% (Fig. 6F and H). Notably, *Ant1* knockdown in BV2 cells resulted in decreased mtDNA leakage into the cytosol following co-exposure to Pb and AngII (Fig. 6I and J). These findings confirm that ANT1 plays an important role in regulating the leakage of mtDNA into the cytoplasm of microglia following Pb and AngII co-exposure.

We further investigated whether ANT1 knockdown could alleviate mitochondrial dysfunction. The results showed that co-exposure to Pb and AngII in BV2 cells led to increased JC-1 and mtSOX levels, opening of the mPTP, and a reduction in ATP content. Notably, *Ant1* knockdown partially reversed these changes in BV2 cells (Figs. S4D to F and S5B).

## Discussion

In the present study, our findings showed that hypertensive mice had increased susceptibility to Pb-induced microglial polarization, exhibiting spatiotemporal dynamic changes, especially in the PFC. Microglial polarization is critically involved in driving neuroinflammatory responses and behavioral dysfunction in hypertensive mice exposed to Pb. Moreover, mtDNA leakage mediates cGAS/STING pathway activity, thereby promoting microglial polarization in hypertensive mice with Pb exposure. Further data demonstrated that ANT1 regulates mtDNA leakage, which mediates cGAS activation. Our findings identify promising therapeutic targets for neuroinflammation in hypertensive individuals and those exposed to Pb.

In addition, the present results reveal that mice following Pb exposure exhibited a significant rise in SBP at 12 weeks, indicating that Pb exposure can elevate the risk of hypertension. Our results were consistent with reports by Sanders et al. [37] and Zhang et al. [38]. Emerging evidence demonstrates that microglia perceive hemodynamic perturbations in the hypertensive state and facilitate sympathetic excitation [39]. In addition, microglia-derived platelet-derived growth factor subunit B has been shown to stabilize neuronal excitability by enhancing potassium currents, thereby counteracting excessive sympathetic activity [40]. Our results demonstrate that M1 microglia and pro-inflammatory cytokine levels were increased in hypertensive mice following Pb exposure. These findings demonstrate that hypertensive mice are more susceptible to Pb-induced microglial M1 polarization and neuroinflammation.

Microglial polarization is a key role regarding neuroinflammation progression [41,42]. The pro-inflammatory M1 type exacerbates nerve injury via cytokine secretion, whereas the M2 type takes a neuroprotective function. Our study demonstrates that an increase in M1 and a decrease in M2 were observed on different weeks in hypertensive mice with Pb exposure, which was accompanied by neuroinflammation and neurobehavioral impairment, suggesting that microglial polarization plays a critical role in nerve injury. In addition, our results showed that the inhibition of microglial polarization (minocycline treatment) significantly attenuates neuroinflammation and depressive-like behaviors, underscoring the dominant role of microglial polarization in Pb- and hypertension-induced neurotoxicity. Our findings parallel observations in diabetic models, where cortical microglia exhibit increased M1 polarization coupled with decreased M2 polarization, collectively driving neuroinflammatory progression [43]. Microglial polarization is a dynamic process [44]. Our results showed that the mice of the Pb group exhibit an increase in M1 polarization at 4 weeks, which is earlier than that of the HTN group. Notably, mice in the Pb+HTN group exhibited a more pronounced and accelerated response where M1 microglia increased significantly as early as 2 weeks, peaked at 12 weeks, and were accompanied by a parallel reduction in M2 microglia. These findings suggest that microglial polarization exhibits temporal specificity in hypertensive mice with Pb exposure. The most profound change in microglial polarization occurred at 12 weeks in the mice the of Pb+HTN group, coinciding with peak neurobehavioral impairment in both the sucrose preference test and FST, suggesting that the 12-week time point may represent a potential inflection point in disease progression. The PFC is one of the primary components of the limbic system, playing an irreplaceable role in emotional responses [45]. Our research findings showed that the most pronounced increases in iNOS and decreases in Arg1, along with elevated IL-1β and IL-6 levels, were observed in the PFC compared to those in the hippocampus and hypothalamus, suggesting that the PFC may be a critical target for Pb- and hypertension-induced neuroinflammation.

There are multiple pathways through which Pb or hypertension can induce microglial polarization [18,46–49]. However, the main pathway remained unclear. High-throughput sequencing of microglia from the Pb, HTN, and Pb+HTN groups, combined with screening through microglial polarization databases, revealed the significant involvement of the cGAS/STING pathway. cGAS/STING signaling has been one of the most well-proven innate immune pathways to be involved in neuroinflammation [50]. Our results showed that inhibiting the cGAS/STING pathway could significantly suppress microglial polarization and improve depressive-like behaviors. These results suggest that the cGAS/STING pathway may represent a key mechanism of microglial polarization, which promotes neuroinflammation in hypertensive mice with Pb exposure. A previous study by Zheng et al. [51] also demonstrated that BV2 cells treated with Aβ polarize into the M1 phenotype, during which the cGAS/STING pathway is activated, which is consistent with our findings. Cytosolic mtDNA serves as one of the triggers in terms of activation of the cGAS/STING pathway [52]. Our findings revealed substantial accumulation of mtDNA within the cytosol of microglia after combined exposure to Pb and AngII, indicating that mtDNA may function as a trigger for cGAS/STING pathway activation associated with microglial polarization. mtDNA serves as a crucial regulator of inflammatory immune processes. Research has demonstrated that reactive oxygen species (ROS) generated by mitochondria can cause oxidative damage to mtDNA, resulting in its subsequent leakage into the cytosol [53]. When present in the cytoplasm, this DNA functions as a damage-associated molecular pattern, stimulating the cGAS/STING/DNA sensing pathway to provoke robust inflammatory reactions. Following activation, the STING protein additionally facilitates the recruitment of TBK1. This kinase then mediates the phosphorylation of IRF3 [54]. In our experiments, the double-stranded DNA of the Pb+AngII group with mitochondrial tracker showed increased leakage of mtDNA into the cytoplasm, accompanied by elevated mtSOX and increased mitochondrial permeability, which suggested that Pb and AngII co-exposure can induce excessive ROS accumulation, promoting mitochondrial damage and mtDNA leakage. Our results are similar to those of studies demonstrating that nanoplastics induce mitochondrial damage, triggering mtDNA leakage and subsequent cGAS/STING activation, ultimately promoting cellular senescence [55].

mtDNA leakage can result from multiple contributing factors. Previous studies have demonstrated that MFN2-mediated mitochondrial fission and fusion promote mtDNA leakage in spinal cord injury [33]. mtDNA can leak from BAX–BAK1 pores and promote hepatocellular carcinoma cell proliferation. Additionally. mPTP opening compromises mitochondrial stability and promotes the release of mtDNA [29]. RNA-seq analysis identified several mitochondrial genes in microglia including *Ant1*, *Nadk2*, *Lig3*, and *Slirp* in terms of mtDNA leakage following Pb and Angll co-exposure. Notably, *Ant1* exhibited the highest fold change among mitochondrial DEGs, suggesting its pivotal role in this process.

ANT1, predominantly situated within the inner mitochondrial membrane, is essential for regulating cellular metabolism. It also contributes to the assembly of the mPTP, a stress-responsive complex whose opening can result in the dissipation of MMP [56–58]. The opening of mPTP allows for the release of various substances, including potentially mtDNA, into the cytosol, which triggers inflammatory responses [59]. Our results showed that the protein expression of ANT1 was significantly increased, whereas inhibiting ANT1 partially restored mtDNA leakage, which suggested that ANT1 is a target for regulating mtDNA leakage following Pb and AngII co-exposure. A study showed that the inhibition of ANT1 can preserve MMP and prevent the subsequent release of mtDNA and ROS, thereby attenuating excessive inflammasome activation [60]. This is similar to our findings, where inhibiting ANT1 reduces mtDNA leakage.

The present study also has limitations. First, our findings have not yet been validated in hypertensive individuals with Pb exposure. Second, although BV2 cells are widely used, they cannot fully recapitulate the complexity of primary microglia and exhibit certain differences in their functions and the magnitude of their responses. Therefore, future studies will employ primary microglia to further investigate the interactions between primary microglia and neurons.

Taken together, an increase in M1 microglia and a decrease in M2 microglia were involved in the development of hypertension with Pb exposure. Notably, microglial polarization presented temporal and spatial characteristics. Meanwhile, Pb and AngII exposure could induce ANT1 expression increase, which promoted mtDNA leakage to cytoplasm. Subsequently, cytosolic mtDNA activated the cGAS/STING signaling pathway, leading to microglial polarization, and then resulted in depressive-like behaviors in hypertensive mice with Pb exposure.

## Materials and Methods

### Animals and treatment

Male C57BL/6J were purchased from Beijing HFK Bio-Technology Co., LTD. The mice were kept in the North China University of Science and Technology Laboratory Animal Center under specific-pathogen-free conditions, with room temperature of 22 ± 3 °C, relative humidity of 40% to 60%, light and darkness of 12 h each, and free diet and water. To establish a hypertensive mice model, half of the mice were intraperitoneally injected with AngII at dose of 0.5 mg/kg/d for 7 d, accompanied by a high-salt diet. An SBP greater than 140 mmHg indicated hypertensive mice. Subsequently, hypertensive mice were randomly divided into the hypertension (HTN) group and Pb + hypertension (Pb+HTN) group, whereas the other half of mice were randomly divided into the control and Pb groups. Furthermore, mice in the Pb or Pb+HTN group were administered with 250 mg/l PbAc drinking water for 2, 4, 8, 12, and 24 weeks, respectively. All animals were approved by the animal ethics committee of the North China University of Science and Technology (No. 2021-SY-200).

For intervention tests, mice were randomly allocated to the control, minocycline (MI), RU.521, Pb+HTN, Pb+HTN+MI, and Pb+HTN+RU.521 groups, each with 15 mice. Pb treatment was the same as the above test. In addition, mice in the MI and Pb+HTN+MI groups were treated with minocycline at 40 mg/kg/d by intraperitoneal injection from 7 until 12 weeks [61]. RU.521(cGAS inhibitor) was administered intranasally (450 μg/kg/d) to mice in the RU.521 group and Pb+HTN+RU.521 group at 12 weeks for 7 d [20,62].

### Behavioral tests

#### Sucrose preference test

The mice were raised separately, given 2 bottles with 1% sucrose, and allowed to drink freely for 24 h, and then one bottle was switched to pure water. After the adaptation, the mice were subjected to fasting and water deprived for 24 h. Next, the mice were given 2 bottles with 1% sucrose or pure water for 24 h, and liquid consumption was recorded. Sucrose preference rate = sucrose consumption/(sucrose consumption + water consumption).

#### Forced swimming test

Mice were put into a cylinder with water and swam freely for 15 min. After 24 h, mice were put into the cylinder again and swam freely for 6 min. The immobility time of the mice in the last 4 min was recorded.

### Cell culture and treatment

BV2 cells were purchased from Procell Life Science & Technology Co., Ltd. BV2 cells were cultured in Dulbecco’s modified Eagle medium (DMEM) with 10% fetal bovine serum and 1% penicillin/streptomycin at 37 °C and 5% CO_2_. For all experimental procedures employing BV2 cell cultures, cellular concentrations were standardized at the following densities: 1 × 10^4^ cells per well when using 96-well culture plates or 5 × 10^5^ cells per well for 6-well plate formats.

### Cell viability assay

Cell viability was evaluated using a colorimetric assay based on Cell Counting Kit-8 (CK04, Dojindo), in accordance with the manufacturer’s instructions. BV2 microglial cells were seeded into 96-well plates at a density of 10,000 cells per well. Upon reaching 80% confluence, the cultures were treated with either PbAc (0, 5, 10, 20, 40, and 80 μmol/l) or AngII (0, 20, 50, 100, and 200 nmol/l) for 24 h. Following treatment, cells were exposed to the Cell Counting Kit-8 reagent for 30 min. Optical density measurements were taken at 450 nm. The viable cells were calculated using the following formula: [(OD_sample_ − OD_blank_)/(OD_control_ − OD_blank_)] × 100.

### Isolation of microglia

Brain tissues were taken from mice after saline perfusion. Then, the brain tissues were digested into cell suspensions and passed through 100- and 40-μm filters and then washed with cold phosphate-buffered saline (PBS). The cells were resuspended in 37% Percoll/DMEM solution and laid over a 70% Percoll/DMEM gradient and then centrifuged at 900 g for 30 min. Cells were harvested from the interface between the 37% and 70% Percoll gradients and subsequently washed with PBS prior to flow cytometric analysis.

### Flow cytometry

Microglia in brain tissue were incubated with CD16/CD32 antibody (4 °C, 30 min). Then, microglia were immunolabeled with fluorescent antibody conjugates targeting specific membrane markers, including anti-CD45 coupled to PerCP-Cy5.5, anti-CD11b conjugated with allophycocyanin, as well as fluorescein isothiocyanate (FITC)-labeled anti-CD86 and phycoerythrin-tagged anti-CD206 antibodies. PBS was utilized to clean the cells 3 times.

BV2 cells are collected after treatment with AngII and Pb. Cells were first treated with CD16/CD32 blocking antibody (30-min incubation at 4 °C) prior to dual-color fluorescence labeling. The samples were subsequently stained with fluorochrome-conjugated antibodies: phycoerythrin-labeled anti-mouse CD206 (eBioscience) and FITC-conjugated anti-mouse CD86 (eBioscience) under light-protected conditions (4 °C, 30 min). Following washing steps with PBS, the cellular expression profiles of M1 (CD86) and M2 (CD206) polarization markers were quantitatively assessed using flow cytometry.

### Western blotting

Brain tissue or cell samples underwent ultrasonic disruption followed by extraction with a complete cell lysis kit (P0013B, Beyotime), strictly following the manufacturer’s protocol. The quantification of protein levels was performed through a bicinchoninic acid assay protocol. The samples were separated through electrophoresis on 10% sodium dodecyl sulfate–polyacrylamide gels and subsequently transferred onto polyvinylidene fluoride membranes. After incubation with 5% nonfat dried milk dissolved in Tris-buffered saline with Tween (TBST) solution to block nonspecific binding sites, the membranes were subjected to overnight incubation at 4 °C with primary antibodies iNOS (1:2,000; A3774, ABclonal), Arg1 (1:1,000; A1847, ABclonal), IL-6 (1:1,000; 26404-1-AP, Proteintech), IL-1β (1:1,000; A22257, ABclonal), cGAS (1:1,000; A8335, ABclonal), STING (1:10,000; A21051, ABclonal), TBK1 (1:1,000; A2573, ABclonal), p-TBK1 (1:1,000; AP1026, ABclonal), IRF3 (1:20,000; 11312-1-AP, Proteintech), p-IRF3 (1:1,000; A2172, ABclonal), and β-actin (1:10,000; AC038, ABclonal). After washing with TBST, the samples were incubated for 2 h at 37 °C with horseradish peroxidase-linked secondary antibodies (AS014 and AS003). Protein bands were detected using an enhanced chemiluminescence plus detection system. To quantify expression levels, the ImageJ software analyzed the grayscale intensities of both the target protein and internal reference bands. Final expression values were derived by calculating the ratio between the target protein and its corresponding reference band.

### Enzyme-linked immunosorbent assay

BV2 microglial cells were seeded into 6-well plates and treated with Pb and AngII for a 24-h period. Subsequently, the culture supernatants were harvested, and the levels of IL-1β and IL-6 in the supernatants were measured using commercially available enzyme-linked immunosorbent assay kits (Solarbio, China) following the manufacturer’s instructions. The assay detection ranges were 7.8 to 500 pg/ml for IL-1β and 18.75 to 1,000 pg/ml for IL-6.

### Real-time PCR

RNA was isolated from BV2 microglial cells using TRIzol reagent (AGbio, China) [15]. Then, the reverse transcription process was performed using a commercially available kit (Zhongshi Tontru, China) to gain complementary DNA. Polymerase chain reaction (PCR) analysis was subsequently performed using Real-Time PCR System (Zhongshi Tontru, China) to evaluate messenger RNA expression levels. For data normalization, β-actin served as the internal control, with relative quantification determined through the 2^−ΔΔCt^ calculation method. The specific primer sequences utilized in these experiments are detailed in Table S3.

### Immunofluorescence

Following established protocols [31,63,64], mice were euthanized under deep anesthesia induced by sodium pentobarbital administration. The brain tissues were separated after transcardial perfusion with PBS, and then the brains were postfixed in 4% paraformaldehyde at 4 °C for 24 h. Subsequently, brain tissues were subjected to cryoprotection through immersion in progressively increasing sucrose concentrations (15% and 30%). The brain specimens were then cryoprotected by embedding in optimal cutting temperature compound medium. Parallel processing involved fixation of BV2 cell cultures in 4% paraformaldehyde solution for 20 min at room temperature. The prepared specimens were utilized for further experimental procedures. Following sequential washes with PBS, which contains 0.4% Triton X-100, both cerebral tissue sections and BV2 cellular preparations underwent blocking with 10% normal goat serum for 50 min. Then, samples were incubated with primary antibodies including rabbit monoclonal anti-cGAS (1:100; A8335, ABclonal), mouse monoclonal anti-Iba-1 (1:100; sc-32725, Santa Cruz Biotechnology), and rabbit monoclonal anti-AT1 (1:100; A15027, ABclonal) overnight at 4 °C. The fluorescently labeled secondary antibody was applied to tissue or cell samples and incubated for 1 h under ambient temperature conditions. Finally, images were obtained using an Olympus FV3000 confocal microscope.

### RNA-seq and analysis

Sample preparation and analysis for RNA-seq were conducted according to our previously published study [65]. Briefly, the total RNA was isolated from harvested cells using TRIzol reagent. Subsequently, complementary DNA libraries were prepared and subjected to RNA-seq analysis at Hangzhou LianChuan Biotechnology Co., Ltd. Gene-level expression was quantified as raw read counts. Differential expression analysis was performed using the limma package in R (version 3.6.1), where raw count data were transformed with the voom method and fitted into a linear model with empirical Bayes moderation to estimate log_2_ fold changes. Genes were considered differentially expressed when exhibiting Log FC values either below −0.5 or above 0.5. To identify genes associated with microglial polarization, the GeneCards database (https://www.genecards.org/) was consulted. The ClusterProfiler package of R was used for Kyoto Encyclopedia of Genes and Genomes analysis.

### siRNA transfection

BV2 cells were plated into 6-well culture plates and underwent transient transfection with either cGAS-targeting small interfering RNA (siRNA) (si-cGAS), ANT1-targeting siRNA (si-ANT1), or nontargeting control siRNA (si-NC) using the HighGene transfection reagent. At 24 h post-transfection, cells were harvested for subsequent experiments.

### Measurement of cytosolic mtDNA

The collected BV2 cells were evenly divided into 2 parts to extract cytoplasmic DNA and total cell DNA, respectively. Total cell DNA was extracted according to the instructions of the cell/tissue genomic DNA extraction kit (DP1902, BioTeke). For extracting cytosolic DNA, the cells were added with 500 μl of digitalin (25 μg/ml) and cleaved for 10 min. The samples underwent centrifugation at 4 °C for 3 min with a relative centrifugal force of 980 g, after which the supernatant obtained was utilized in subsequent analytical procedures. Then, cytosolic mtDNA was extracted by using a DNA extraction kit (DP1902, BioTeke, China). The expression of mitochondrial genes including *D-loop1*, *ND1*, and *D-loop3* in cytoplasmic DNA was detected by quantitative PCR. Tert from total DNA was used as an internal reference to assess the expression of target genes. The primer sequences are presented in Table S4.

### mtDNA depletion in BV2 cells

For mtDNA depletion, BV2 cells were cultured with 0.2 μg/ml EtBr for 2 d. DNA was extracted from the cell with a genomic DNA extraction kit (DP1902, BioTeke, China). The mtDNA content was evaluated using quantitative PCR as described above.

### MMP measurement

JC-1 Assay Kit (Beyotime) was used to assess MMP. Following PBS washes, cellular samples were treated with JC-1 working solution at a 1:2 dilution. Fluorescence imaging was subsequently performed with Olympus FV3000.

### ATP measurement

Cells were lysed in detergent to inactivate ATPase and centrifuged (12,000 g, 4 °C for 5 min), and the supernatant was collected. The supernatant was used to determine the total ATP protein concentration. Assay buffer was added to each supernatant. After incubation, luminescence was measured in a luminometer.

### Mitochondrial SOX assay

The testing of MitoSOX was carried out following the instructions (MT14, Dojindo, Japan). BV2 cells were added with 10 μM MitoSOX solution and incubated at 37 °C for 30 min. Subsequently, the cellular samples underwent 3 washing cycles using PBS before being resuspended in fresh PBS solution. Mitochondrial SOX levels were then quantified through flow cytometric analysis.

### Measurement of the mPTP

The opening of the mPTP was evaluated using an mPTP assay kit (BL928A, Biosharp). Briefly, cells from each group were rinsed with PBS and incubated at 37 °C for 30 min with calcein-AM in the presence of cobalt chloride (Co^2+^) as a quencher. After incubation, the staining solution was replaced with fresh culture medium, and the cells were maintained at 37 °C in the dark for an additional 30 min. Flow cytometry was used to measure fluorescence intensity.

### Statistical analysis

Data are expressed as mean ± SD (*$\bar{x}$* ± *s*). For statistical analysis, version 23.0 of SPSS was utilized. For intergroup comparisons, we employed a one-way analysis of variance approach with subsequent least significant difference *t* testing to identify specific differences. A threshold of *P* < 0.05 denoted statistical significance across all analyses.

## Data Availability

Data will be made available on request.

## Supplementary Materials

Supplementary 1 Figs. S1 to S5 Tables S1 to S4 Supplementary Materials 2—Blots

## References

[1] Kario K, Okura A, Hoshide S, Mogi M. The WHO Global report 2023 on hypertension warning the emerging hypertension burden in globe and its treatment strategy. Hypertens Res. 2024;47(5):1099–1102. 38443614 10.1038/s41440-024-01622-w

[2] Muela HC, Costa-Hong VA, Yassuda MS, Moraes NC, Memoria CM, Machado MF, Macedo TA, Shu EB, Massaro AR, Nitrini R, et al. Hypertension severity is associated with impaired cognitive performance. J Am Heart Assoc. 2017;6(1):1014–1030. 10.1161/JAHA.116.004579 PMC5523638 28077386

[3] Youwakim J, Girouard H. Inflammation: A mediator between hypertension and neurodegenerative diseases. Am J Hypertens. 2021;34(10):1014–1030. 34136907 10.1093/ajh/hpab094

[4] Pan Z, Gong T, Liang P. Heavy metal exposure and cardiovascular disease. Circ Res. 2024;134(9):1160–1178. 38662861 10.1161/CIRCRESAHA.123.323617

[5] Lamas GA, Bhatnagar A, Jones MR, Mann KK, Nasir K, Tellez-Plaza M, Ujueta F, Navas-Acien A. Contaminant metals as cardiovascular risk factors: A scientific statement from the American Heart Association. J Am Heart Assoc. 2023;12(13): Article e29852. 10.1161/JAHA.123.029852 PMC10356104 37306302

[6] Wildemann TM, Mirhosseini N, Siciliano SD, Weber LP. Cardiovascular responses to lead are biphasic, while methylmercury, but not inorganic mercury, monotonically increases blood pressure in rats. Toxicology. 2015;328:1–11. 25478804 10.1016/j.tox.2014.11.009

[7] Afridi HI, Brabazon D, Kazi TG, Naher S, Nesterenko E. Comparative metal distribution in scalp hair of Pakistani and Irish referents and hypertensive patients. Biol Trace Elem Res. 2011;143(3):1367–1382. 21301989 10.1007/s12011-011-8985-1

[8] Bajwa E, Klegeris A. Neuroinflammation as a mechanism linking hypertension with the increased risk of Alzheimer’s disease. Neural Regen Res. 2022;17(11):2342–2346. 35535868 10.4103/1673-5374.336869 PMC9120695

[9] Wang J, Zhang W, Xu H, Ellenbroek B, Dai J, Wang L, Yan C, Wang W. The changes of histone methylation induced by adolescent social stress regulate the resting-state activity in mPFC. Research. 2023;6:264. 10.34133/research.0264 PMC10907022 38434244

[10] Mirzac D, Kreis SL, Luhmann HJ, Gonzalez-Escamilla G, Groppa S. Translating pathological brain activity primers in Parkinson’s disease research. Research. 2023;6:183. 10.34133/research.0183 PMC10298229 37383218

[11] Cao L, He C. Polarization of macrophages and microglia in inflammatory demyelination. Neurosci Bull. 2013;29(2):189–198. 23558588 10.1007/s12264-013-1324-0 PMC5561884

[12] David S, Kroner A. Repertoire of microglial and macrophage responses after spinal cord injury. Nat Rev Neurosci. 2011;12(7):388–399. 21673720 10.1038/nrn3053

[13] Durafourt BA, Moore CS, Zammit DA, Johnson TA, Zaguia F, Guiot MC, Bar-Or A, Antel JP. Comparison of polarization properties of human adult microglia and blood-derived macrophages. Glia. 2012;60(5):717–727. 22290798 10.1002/glia.22298

[14] Franco R, Fernandez-Suarez D. Alternatively activated microglia and macrophages in the central nervous system. Prog Neurobiol. 2015;131:65–86. 26067058 10.1016/j.pneurobio.2015.05.003

[15] Wang L, Liu T, Wang X, Tong L, Chen G, Zhou S, Zhang H, Liu H, Lu W, Wang G, et al. Microglia-derived TNF-α contributes to RVLM neuronal mitochondrial dysfunction via blocking the AMPK–Sirt3 pathway in stress-induced hypertension. J Neuroinflammation. 2023;20(1):137. 37264405 10.1186/s12974-023-02818-6 PMC10236846

[16] Liu MC, Liu XQ, Wang W, Shen XF, Che HL, Guo YY, Zhao MG, Chen JY, Luo WJ. Involvement of microglia activation in the lead induced long-term potentiation impairment. PLOS ONE. 2012;7(8): Article e43924. 22952811 10.1371/journal.pone.0043924 PMC3432044

[17] Gassowska-Dobrowolska M, Chlubek M, Kolasa A, Tomasiak P, Korbecki J, Skowronska K, Tarnowski M, Masztalewicz M, Baranowska-Bosiacka I. Microglia and astroglia—The potential role in neuroinflammation induced by pre- and neonatal exposure to lead (Pb). Int J Mol Sci. 2023;24(12):9903. 37373050 10.3390/ijms24129903 PMC10298497

[18] Su P, Zhang J, Wu J, Chen H, Luo W, Hu M. TREM2 expression on the microglia resolved lead exposure-induced neuroinflammation by promoting anti-inflammatory activities. Ecotoxicol Environ Saf. 2023;260: Article 115058. 37245276 10.1016/j.ecoenv.2023.115058

[19] Mao H, Angelini A, Li S, Wang G, Li L, Patterson C, Pi X, Xie L. CRAT links cholesterol metabolism to innate immune responses in the heart. Nat Metab. 2023;5(8):1382–1394. 37443356 10.1038/s42255-023-00844-5 PMC10685850

[20] Shao J, Meng Y, Yuan K, Wu Q, Zhu S, Li Y, Wu P, Zheng J, Shi H. RU.521 mitigates subarachnoid hemorrhage-induced brain injury via regulating microglial polarization and neuroinflammation mediated by the cGAS/STING/NF-κB pathway. Cell Commun Signal. 2023;21(1):264. 37770901 10.1186/s12964-023-01274-2 PMC10537158

[21] Wu W, Zhang X, Wang S, Li T, Hao Q, Li S, Yao W, Sun R. Pharmacological inhibition of the cGAS-STING signaling pathway suppresses microglial M1-polarization in the spinal cord and attenuates neuropathic pain. Neuropharmacology. 2022;217: Article 109206. 35926582 10.1016/j.neuropharm.2022.109206

[22] Riley JS, Tait SW. Mitochondrial DNA in inflammation and immunity. EMBO Rep. 2020;21(4): Article e49799. 32202065 10.15252/embr.201949799 PMC7132203

[23] Brooks N, Riar I, Klegeris A. Mitochondrial damage-associated molecular patterns: Neuroimmunomodulators in central nervous system pathophysiology. Neural Regen Res. 2026;21(4):1322–1338. 40537002 10.4103/NRR.NRR-D-24-01459 PMC12407514

[24] Suptela AJ, Marriott I. Cytosolic DNA sensors and glial responses to endogenous DNA. Front Immunol. 2023;14:1130172. 36999037 10.3389/fimmu.2023.1130172 PMC10043442

[25] Pan P, Cao S, Gao H, Qu X, Ma Y, Yang J, Pei X, Yang Y. Immp2l gene knockout induces granulosa cell senescence by activation of cGAS-STING pathway via TFAM-mediated mtDNA leakage. Int J Biol Macromol. 2025;307(Pt 4): Article 142368. 40120895 10.1016/j.ijbiomac.2025.142368

[26] Shadel GS, Horvath TL. Mitochondrial ROS signaling in organismal homeostasis. Cell. 2015;163(3):560–569. 26496603 10.1016/j.cell.2015.10.001 PMC4634671

[27] Mani MS, Chakrabarty S, Mallya SP, Kabekkodu SP, Jayaram P, Varghese VK, Dsouza HS, Satyamoorthy K. Whole mitochondria genome mutational spectrum in occupationally exposed lead subjects. Mitochondrion. 2019;48:60–66. 31029642 10.1016/j.mito.2019.04.009

[28] McCarthy CG, Wenceslau CF, Goulopoulou S, Ogbi S, Baban B, Sullivan JC, Matsumoto T, Webb RC. Circulating mitochondrial DNA and Toll-like receptor 9 are associated with vascular dysfunction in spontaneously hypertensive rats. Cardiovasc Res. 2015;107(1):119–130. 25910936 10.1093/cvr/cvv137 PMC4560046

[29] Newman LE, Shadel GS. Mitochondrial DNA release in innate immune signaling. Annu Rev Biochem. 2023;92:299–332. 37001140 10.1146/annurev-biochem-032620-104401 PMC11058562

[30] Zou R, Tao J, He J, Wang C, Tan S, Xia Y, Chang X, Li R, Wang G, Zhou H, et al. PGAM5-mediated PHB2 dephosphorylation contributes to diabetic cardiomyopathy by disrupting mitochondrial quality surveillance. Research. 2022;2022:0001. 39285950 10.34133/research.0001 PMC11404314

[31] Dao FY, Lv H, Fullwood MJ, Lin H. Accurate identification of DNA replication origin by fusing epigenomics and chromatin interaction information. Research. 2022;2022:9780293. 36405252 10.34133/2022/9780293 PMC9667886

[32] Wang Y, Xu X, Jiang G. Microplastics exposure promotes the proliferation of skin cancer cells but inhibits the growth of normal skin cells by regulating the inflammatory process. Ecotoxicol Environ Saf. 2023;267: Article 115636. 37918331 10.1016/j.ecoenv.2023.115636

[33] Wei FL, Wang TF, Wang CL, Zhang ZP, Zhao JW, Heng W, Tang Z, Du MR, Yan XD, Li XX, et al. Cytoplasmic escape of mitochondrial DNA mediated by Mfn2 downregulation promotes microglial activation via cGas-Sting axis in spinal cord injury. Adv Sci. 2024;11(4): Article e2305442. 10.1002/advs.202305442 PMC10811505 38009491

[34] Lu Y, Zhou M, Li Y, Li Y, Hua Y, Fan Y. Minocycline promotes functional recovery in ischemic stroke by modulating microglia polarization through STAT1/STAT6 pathways. Biochem Pharmacol. 2021;186: Article 114464. 33577892 10.1016/j.bcp.2021.114464

[35] Huang LS, Hong Z, Wu W, Xiong S, Zhong M, Gao X, Rehman J, Malik AB. mtDNA activates cGAS signaling and suppresses the YAP-mediated endothelial cell proliferation program to promote inflammatory injury. Immunity. 2020;52(3):475–486. 32164878 10.1016/j.immuni.2020.02.002 PMC7266657

[36] Ouyang W, Wang S, Yan D, Wu J, Zhang Y, Li W, Hu J, Liu Z. The cGAS-STING pathway-dependent sensing of mitochondrial DNA mediates ocular surface inflammation. Signal Transduct Target Ther. 2023;8(1):371. 37735446 10.1038/s41392-023-01624-z PMC10514335

[37] Sanders AP, Mazzella MJ, Malin AJ, Hair GM, Busgang SA, Saland JM, Curtin P. Combined exposure to lead, cadmium, mercury, and arsenic and kidney health in adolescents age 12–19 in NHANES 2009–2014. Environ Int. 2019;131: Article 104993. 31326826 10.1016/j.envint.2019.104993 PMC6750805

[38] Zhang R, Zhou J, Huo P, Zhang H, Shen H, Huang Q, Chen G, Yang L, Zhang D. Exposure to multiple metal(loid)s and hypertension in Chinese older adults. Biol Trace Elem Res. 2025;203(6):2944–2959. 39320571 10.1007/s12011-024-04388-x

[39] Wei B, Cheng G, Bi Q, Lu C, Sun Q, Li L, Chen N, Hu M, Lu H, Xu X, et al. Microglia in the hypothalamic paraventricular nucleus sense hemodynamic disturbance and promote sympathetic excitation in hypertension. Immunity. 2024;57(9):2030–2042. 39116878 10.1016/j.immuni.2024.07.011

[40] Bi Q, Wang C, Cheng G, Chen N, Wei B, Liu X, Li L, Lu C, He J, Weng Y, et al. Microglia-derived PDGFB promotes neuronal potassium currents to suppress basal sympathetic tonicity and limit hypertension. Immunity. 2022;55(8):1466–1482. 35863346 10.1016/j.immuni.2022.06.018

[41] Xu L, He D, Bai Y. Microglia-mediated inflammation and neurodegenerative disease. Mol Neurobiol. 2016;53(10):6709–6715. 26659872 10.1007/s12035-015-9593-4

[42] Hoogland IC, Houbolt C, van Westerloo DJ, van Gool WA, van de Beek D. Systemic inflammation and microglial activation: Systematic review of animal experiments. J Neuroinflammation. 2015;12:114. 26048578 10.1186/s12974-015-0332-6 PMC4470063

[43] Huang Y, Liao Z, Lin X, Wu X, Chen X, Bai X, Zhuang Y, Yang Y, Zhang J. Overexpression of miR-146a might regulate polarization transitions of BV-2 cells induced by high glucose and glucose fluctuations. Front Endocrinol. 2019;10:719. 10.3389/fendo.2019.00719 PMC6817609 31695681

[44] Lang GP, Li C, Han YY. Rutin pretreatment promotes microglial M1 to M2 phenotype polarization. Neural Regen Res. 2021;16(12):2499–2504. 33907040 10.4103/1673-5374.313050 PMC8374565

[45] Le Merre P, Ahrlund-Richter S, Carlen M. The mouse prefrontal cortex: Unity in diversity. Neuron. 2021;109(12):1925–1944. 33894133 10.1016/j.neuron.2021.03.035

[46] Xue B, Gumusoglu SB, Tiarks G, Todd BP, Wong A, Santillan DA, Kuo CC, Chiang HY, Ravindranath R, Wang SY, et al. Gestational hypertension increases risk of seizures in children and mice. J Clin Invest. 2025;135(12): Article e183393. 40519163 10.1172/JCI183393 PMC12165801

[47] Yu Y, Xue B, Tong L, Bassuk AG, Johnson AK, Wei SG. RORγt mediates angiotensin II-induced pressor responses, microglia activation, and neuroinflammation by disrupting the blood–brain barrier in rats. J Am Heart Assoc. 2025;14(5): Article e40461. 10.1161/JAHA.124.040461 PMC12132782 40008506

[48] Tang W, Peng J, Chen L, Yu C, Wang Y, Zou F, Zheng G, Meng X. Lead inhibits microglial cell migration via suppression of store-operated calcium entry. Toxicol Lett. 2024;393:69–77. 38281554 10.1016/j.toxlet.2024.01.011

[49] Jiang C, Li X, Xiang C, Ye F. Pb induces the release of CXCL10 and CCL2 chemokines via mtROS/NF-κB activation in BV-2 cells. Toxicol Lett. 2024;391:62–70. 38061439 10.1016/j.toxlet.2023.12.001

[50] Jin X, Wang W, Zhao X, Jiang W, Shao Q, Chen Z, Huang C. The battle between the innate immune cGAS-STING signaling pathway and human herpesvirus infection. Front Immunol. 2023;14:1235590. 37600809 10.3389/fimmu.2023.1235590 PMC10433641

[51] Wu Z, Tang W, Ibrahim F, Chen X, Yan H, Tao C, Wang Z, Guo Y, Fu Y, Wang Q, et al. Aβ induces neuroinflammation and microglial M1 polarization via cGAS-STING-IFITM3 signaling pathway in BV-2 cells. Neurochem Res. 2023;48(9):2881–2894. 37210413 10.1007/s11064-023-03945-5

[52] Motwani M, Pesiridis S, Fitzgerald KA. DNA sensing by the cGAS–STING pathway in health and disease. Nat Rev Genet. 2019;20(11):657–674. 31358977 10.1038/s41576-019-0151-1

[53] Zhao M, Wang Y, Li L, Liu S, Wang C, Yuan Y, Yang G, Chen Y, Cheng J, Lu Y, et al. Mitochondrial ROS promote mitochondrial dysfunction and inflammation in ischemic acute kidney injury by disrupting TFAM-mediated mtDNA maintenance. Theranostics. 2021;11(4):1845–1863. 33408785 10.7150/thno.50905 PMC7778599

[54] Arumugam S, Li B, Boodapati S, Nathanson MH, Sun B, Ouyang X, Mehal WZ. Mitochondrial DNA and the STING pathway are required for hepatic stellate cell activation. Hepatology. 2023;78(5):1448–1461. 37013923 10.1097/HEP.0000000000000388 PMC10804318

[55] Wang K, Du Y, Li P, Guan C, Zhou M, Wu L, Liu Z, Huang Z. Nanoplastics causes heart aging/myocardial cell senescence through the Ca^2+^/mtDNA/cGAS-STING signaling cascade. J Nanobiotechnology. 2024;22(1):96. 38448951 10.1186/s12951-024-02375-x PMC10918962

[56] Tang X, Zhao S, Liu J, Liu X, Sha X, Huang C, Hu L, Sun S, Gao Y, Chen H, et al. Mitochondrial GSNOR alleviates cardiac dysfunction via ANT1 denitrosylation. Circ Res. 2023;133(3):220–236. 37377022 10.1161/CIRCRESAHA.123.322654

[57] Nakahara K, Tanaka T, Okuda H, Isonishi A, Morita-Takemura S, Tatsumi K, Wanaka A. The inner mitochondrial membrane protein ANT1 modulates IL-6 expression via the JNK pathway in macrophages. FEBS Lett. 2018;592(22):3750–3758. 30311946 10.1002/1873-3468.13269

[58] Liu J, Ding W, Chen Q, Peng Y, Kong Y, Ma L, Zhang W. Adenine nucleotide translocase 1 promotes functional integrity of mitochondria via activating DDIT3-CytC pathway and intensifying actin filament structures. Mol Neurobiol. 2025;62(7):8457–8474. 40011359 10.1007/s12035-025-04710-1

[59] Patel P, Mendoza A, Ramirez D, Robichaux D, Molkentin JD, Karch J. The adenine nucleotide translocase family underlies cardiac ischemia-reperfusion injury through the mitochondrial permeability pore independently of cyclophilin D. Sci Adv. 2024;10(50): Article p7444. 10.1126/sciadv.adp7444 PMC11633734 39661674

[60] Guo W, Liu W, Chen Z, Gu Y, Peng S, Shen L, Shen Y, Wang X, Feng GS, Sun Y, et al. Tyrosine phosphatase SHP2 negatively regulates NLRP3 inflammasome activation via ANT1-dependent mitochondrial homeostasis. Nat Commun. 2017;8(1):2168. 29255148 10.1038/s41467-017-02351-0 PMC5735095

[61] Liu X, Chen Y, Wang H, Wei Y, Yuan Y, Zhou Q, Fang F, Shi S, Jiang X, Dong Y, et al. Microglia-derived IL-1β promoted neuronal apoptosis through ER stress-mediated signaling pathway PERK/eIF2α/ATF4/CHOP upon arsenic exposure. J Hazard Mater. 2021;417: Article 125997. 34229406 10.1016/j.jhazmat.2021.125997

[62] Ding R, Li H, Liu Y, Ou W, Zhang X, Chai H, Huang X, Yang W, Wang Q. Activating cGAS–STING axis contributes to neuroinflammation in CVST mouse model and induces inflammasome activation and microglia pyroptosis. J Neuroinflammation. 2022;19(1):137. 35689216 10.1186/s12974-022-02511-0 PMC9188164

[63] Zhang Y, Wang L, Pan Q, Yang X, Cao Y, Yan J, Wang Y, Tao Y, Fan R, Sun X, et al. Selective sphingosine-1-phosphate receptor 1 modulator attenuates blood–brain barrier disruption following traumatic brain injury by inhibiting vesicular transcytosis. Fluids Barriers CNS. 2022;19(1):57. 35820896 10.1186/s12987-022-00356-6 PMC9277863

[64] Li J, Karakas D, Xue F, Chen Y, Zhu G, Yucel YH, MacParland SA, Zhang H, Semple JW, Freedman J, et al. Desialylated platelet clearance in the liver is a novel mechanism of systemic immunosuppression. Research. 2023;6:236. 10.34133/research.0236 PMC10551749 37808178

[65] Shi F, Yang H, Sun G, Cui J, Li Z, Wang W, Zhang Y. Pb induces ferroptosis in choroid plexus epithelial cells via Fe metabolism. Neurotoxicology. 2023;95:107–116. 36642386 10.1016/j.neuro.2023.01.005

