# Supplementary material for: Hypertension Increases Susceptibility to Lead-Induced Microglial Polarization via ANT1-Mediated Mitochondrial DNA/cGAS/STING Signaling
Source: Research (Wash D C). 2025 Dec 15;8:1026. doi: 10.34133/research.1026 (PMC12703018; doi:10.34133/research.1026)
Supplement: Supplementary 1 — Figs. S1 to S5 Tables S1 to S4 Supplementary Materials 2—Blots [file research.1026.f1.zip › Supplemental material-1.docx]

**Supplementary materials**

This supporting information includes:

Supplementary Figures S1 to S5

Supplementary Table S1 to S4

**
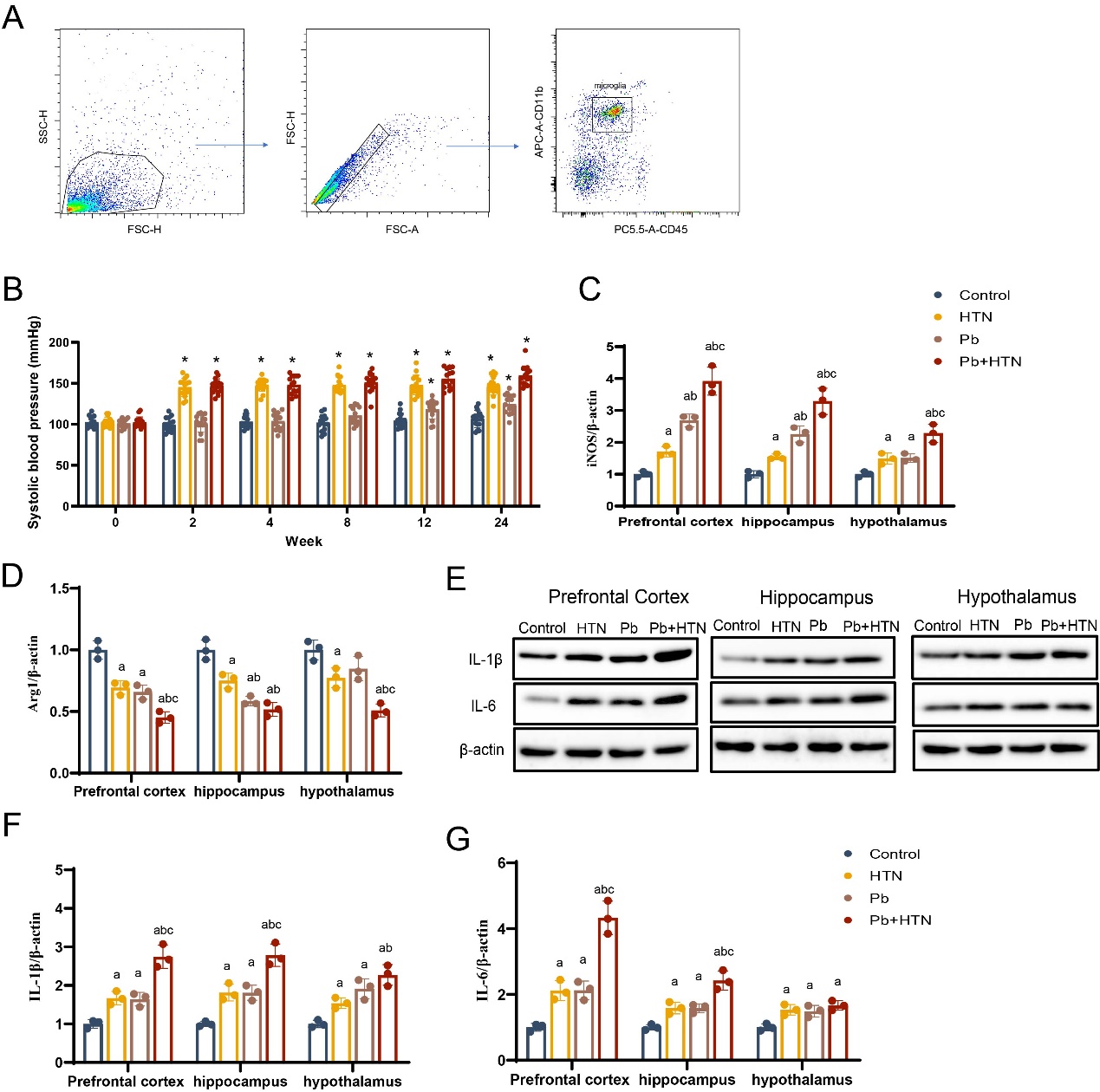
**

**SFig.1 Blood pressure and microglial inflammatory cytokine changes in Pb-exposed hypertensive mice.** (A) Flow cytometry analysis showing the percentage of microglia (CD11b^+^CD45^+^) (n=6). (B) Systolic blood pressure. (C and D) Relative gray values of iNOS and Arg1 proteins. (E) WB showing the expression of IL-1β and IL-6 in the prefrontal cortex, hippocampus and hypothalamus of each group mice (n=3). (F and G) Relative gray values of IL-1β and IL-6 proteins. ^a^*P* < 0.05 vs control group; ^b^*P* < 0.05 vs HTN group; ^c^*P* < 0.05 vs Pb group.

**
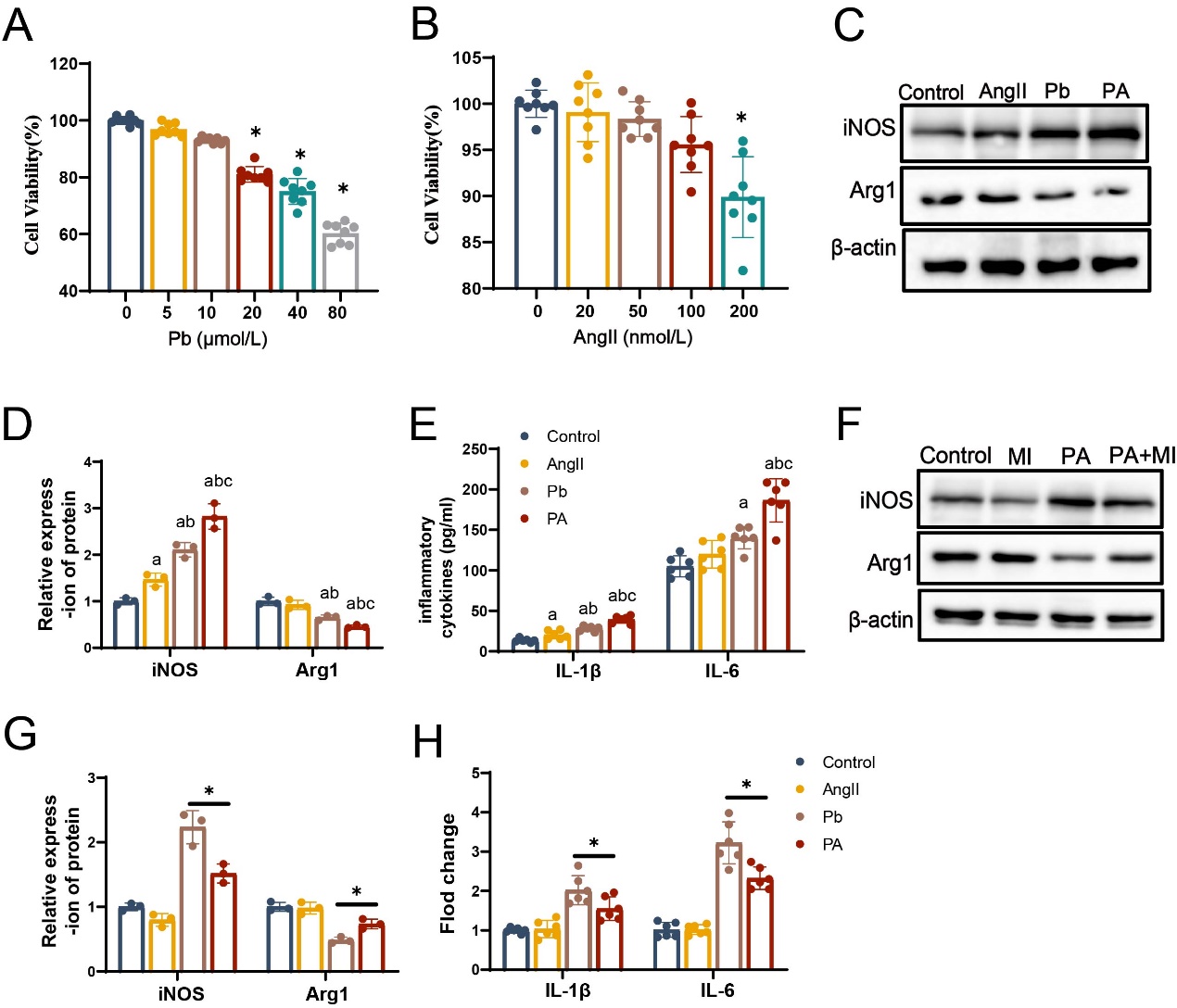
**

**SFig. 2 Effect of minocycline on BV2 polarization induced by Pb and AngII co-exposure.** (A and B) The survival rate of BV2 was detected by CCK8 assay (n=8). (B) WB showing the expression of iNOS and Arg1 in BV2 cells (n=3). (D) Relative gray values of iNOS and Arg1 proteins. (E) The mRNA expression of IL-1β and IL-6 in different groups (n=6). (F) WB showing the expression of iNOS and Arg1 in BV2 cells treated with minocycline (n=3). (G) Relative gray values of iNOS and Arg1 proteins. (H) The mRNA expression of IL-1β and IL-6 in different groups (n=6). ^a^*P* < 0.05 vs control group; ^b^*P* < 0.05 vs HTN group; ^c^*P* < 0.05 vs Pb group; ^*^*P* < 0.05.

**
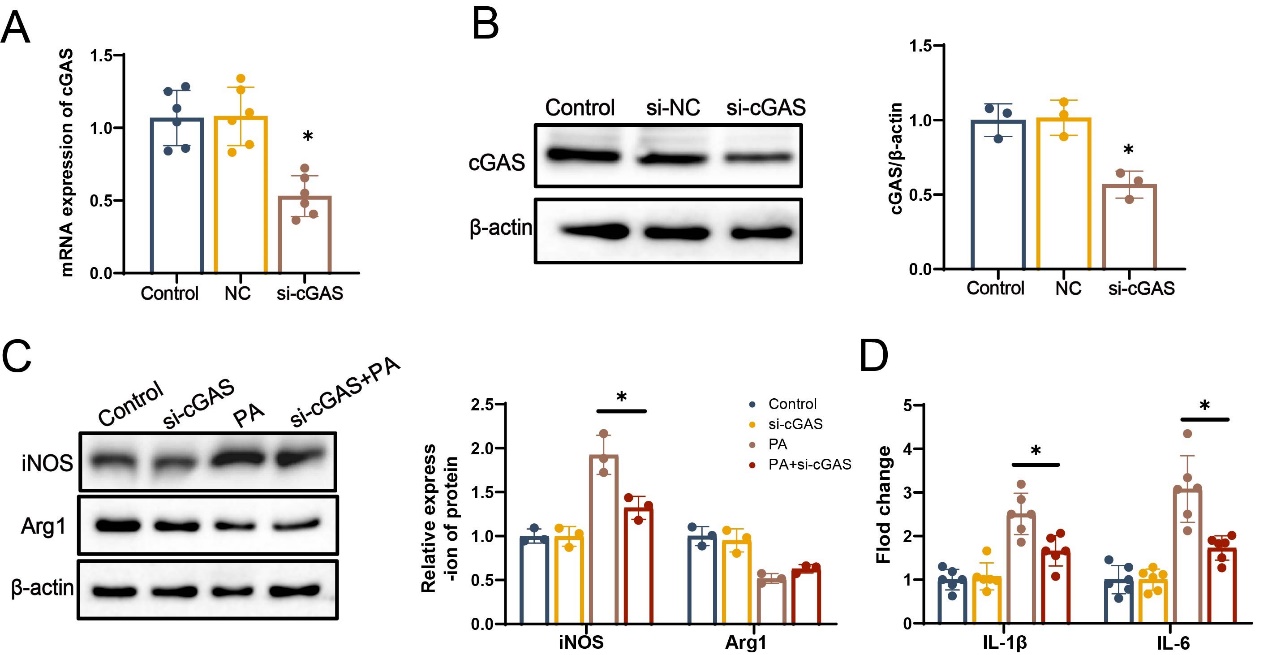
**

**SFig. 3 Effect of cGAS on BV2 polarization induced by Pb and AngII co-exposure.** (A) The mRNA expression of cGAS in BV2 cells (n=6). (B) WB showing the expression of cGAS in BV2 cells (n=3). (C) WB showing the expression of iNOS and Arg1 in BV2 cells. (D) The mRNA expression of IL-1β and IL-6 in different groups (n=6). ^*^*P* < 0.05.

**
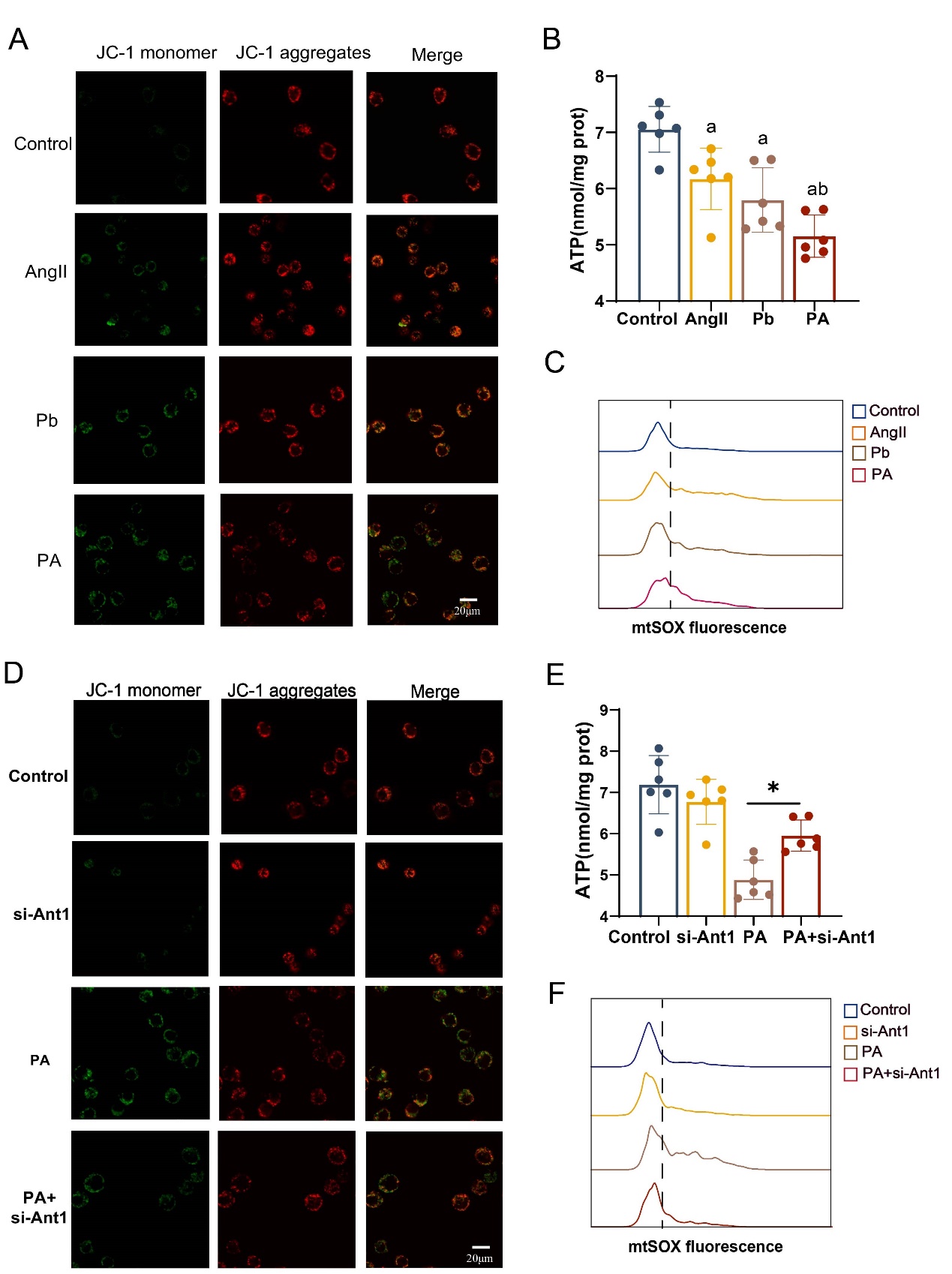
**

**SFig. 4 Mitochondrial damage status in microglia from different groups.** (A-C) MMP levels, ATP content and mtSOX detected by JC-1 fluorescence, flow cytometry and ATP Assay Kit (n=6). (D-F) MMP levels, ATP content and mtSOX detected by JC-1 fluorescence, flow cytometry and ATP Assay Kit (n=6). ^a^*P* < 0.05 vs control group; ^b^*P* < 0.05 vs AngII group; ^c^*P* < 0.05 vs Pb group ^*^*P* < 0.05.

**
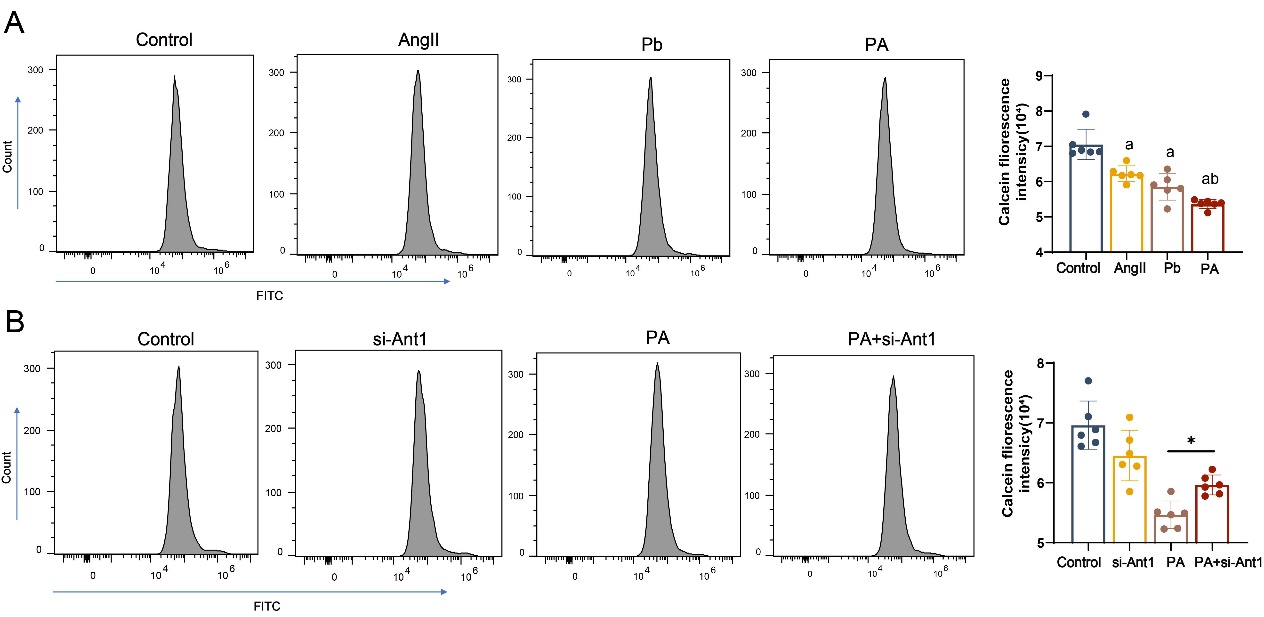
**

**SFig. 5 Mitochondrial mPTP opening in microglia from different groups.** (A, B) Representative fluorescence analyses of calcein AM/Co^2+^ quencher staining and the quantitative analysis of fluorescence intensity for opening levels of mPTP in BV2 cells (n=6). ^a^*P* < 0.05 vs control group; ^b^*P* < 0.05 vs AngII group; ^c^*P* < 0.05 vs Pb group ^*^*P* < 0.05.

**Supplementary Table S1. M1-type microglia percentage in brain tissue of Pb-exposed hypertensive mice**

| Week | Control | HTN | Pb | Pb+HTN |
| --- | --- | --- | --- | --- |
| 2W | 4.90±0.49 | 5.20±0.50 | 5.16±0.56 | 5.95±0.75^a^ |
| 4W | 4.78±0.58 | 5.6±0.48 | 6.05±0.63^a^ | 6.46±0.54^a^ |
| 8W | 4.33±0.45 | 5.59±0.67^a^ | 7.04±0.66^ab^ | 9.62±0.74^abc^ |
| 12W | 5.09±0.90 | 9.86±1.03^a^ | 14.02±1.48^ab^ | 17.53±2.58^abc^ |
| 24W | 6.16±0.58 | 9.15±0.92^a^ | 15.12±1.32^ab^ | 20.5±2.01^abc^ |

^a^*P* < 0.05 vs control group; ^b^*P* < 0.05 vs HTN group; ^c^*P* < 0.05 vs Pb group.

**Supplementary Table S2. M2-type microglia percentage in brain tissue of Pb-exposed hypertensive mice**

| Week | Control | HTN | Pb | Pb+HTN |
| --- | --- | --- | --- | --- |
| 2W | 14.56±1.13 | 13.62±1.27 | 15.06±1.85 | 15.48±1.51 |
| 4W | 14.77±1.60 | 14.65±1.32 | 12.31±1.24^ab^ | 11.04±1.22^ab^ |
| 8W | 14.77±1.12 | 13.55±1.05 | 11.82±0.96^ab^ | 10.97±1.22^ab^ |
| 12W | 14.48±1.25 | 12.37±1.11^a^ | 9.43±0.97^ab^ | 7.21±0.89^abc^ |
| 24W | 16.43±1.48 | 12.17±1.32^a^ | 9.47±0.91^ab^ | 8.58±0.90^ab^ |

^a^*P* < 0.05 vs control group; ^b^*P* < 0.05 vs HTN group; ^c^*P* < 0.05 vs Pb group.

**Supplementary Table S3. The primer sequences of IL-1β, IL-6, cGAS, Ant1,** **β-actin**

| Gene | Forward primer | Reverse primer |
| --- | --- | --- |
| IL-1β | GGCAACTGTTCCTGAACTCAACTG | CCATTGAGGTGGAGAGCTTTCAGC |
| IL-6 | CTCCCAACAGACCTGTCTATAC | CCATTGCACAACTCTTTTCTCA |
| cGAS | GAGGCGCGGAAAGTCGTAA | GAGGCGCGGAAAGTCGTAA |
| IFN-β | CCAGTTCCGACAAAGCACTA | GAATGGCAAAGGCAGTGTAAC |
| Ant1 | GCTCTTACTGACTGGCATGAG | CGCAGCTCTAGGAGCATGTG |
| β-actin | GTTGGTTGGAGCAAACATCCC | TTAGGAGTGGGGGTGGCTTT |

**Supplementary Table S4 The primer sequences of Dloop1, Dloop3, ND1 and** **Tert**

| Gene | Forward primer | Reverse primer |
| --- | --- | --- |
| Dloop1 | AATCTACCATCCTCCGTGAAACC | TCAGTTTAGCTACCCCCAAGTTTAA |
| Dloop3 | TCCTCCGTGAAACCAACAA | AGCGAGAAGAGGGGCATT |
| ND1 | CAAACACTTATTACAACCCAAGAACA | TCATATTATGGCTATGGGTCAGG |
| Tert | CTAGCTCATGTGTCAAGACCCTCTT | GCCAGCACGTTTCTCTCGTT |
