# Supplementary figures and images for: Hypertension Increases Susceptibility to Lead-Induced Microglial Polarization via ANT1-Mediated Mitochondrial DNA/cGAS/STING Signaling
Source: Research (Wash D C). 2025 Dec 15;8:1026. doi: 10.34133/research.1026 (PMC12703018; doi:10.34133/research.1026)

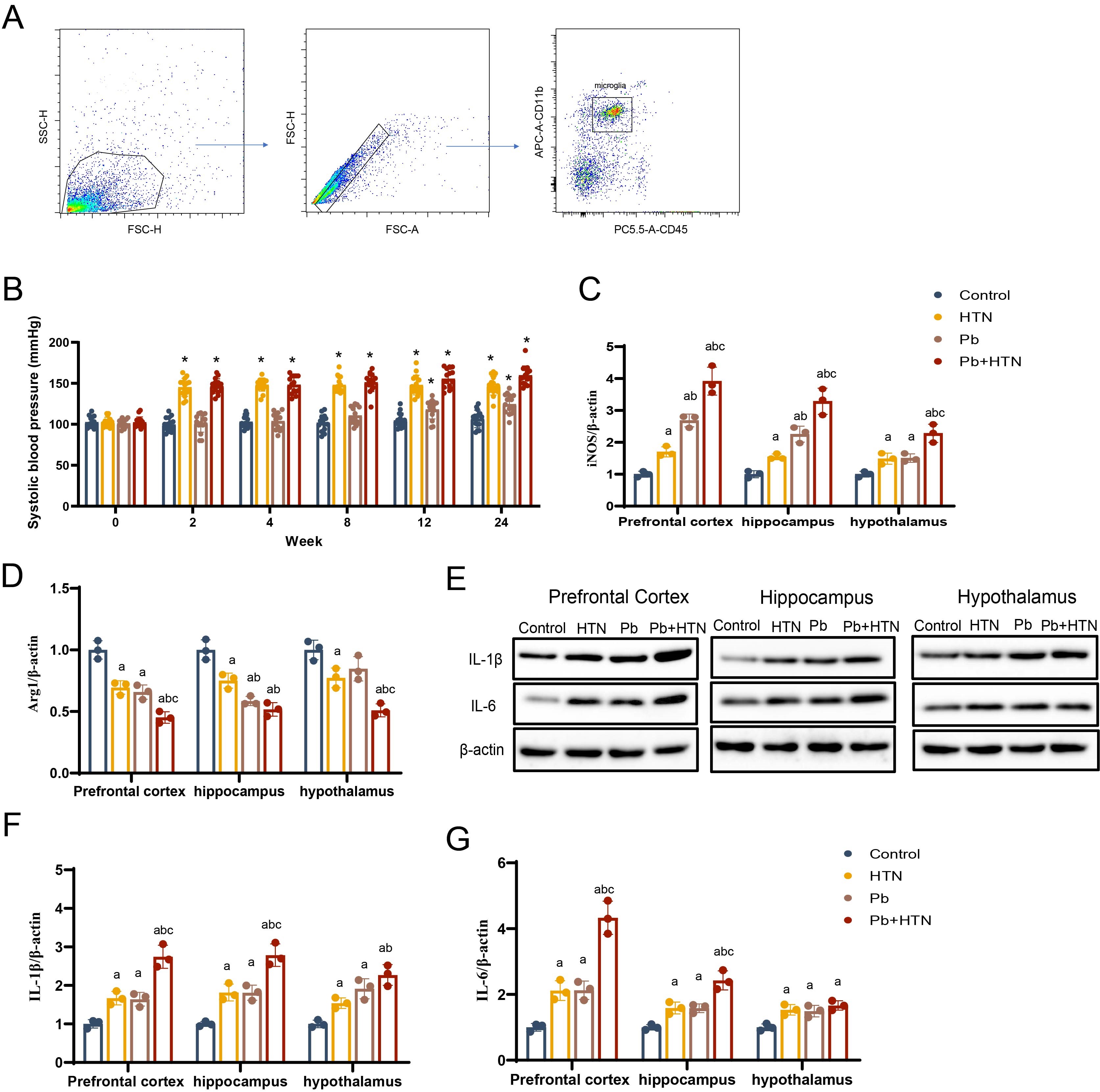

Supplement: Supplementary 1 — Figs. S1 to S5 Tables S1 to S4 Supplementary Materials 2—Blots [file research.1026.f1.zip › FIGS1.jpeg]

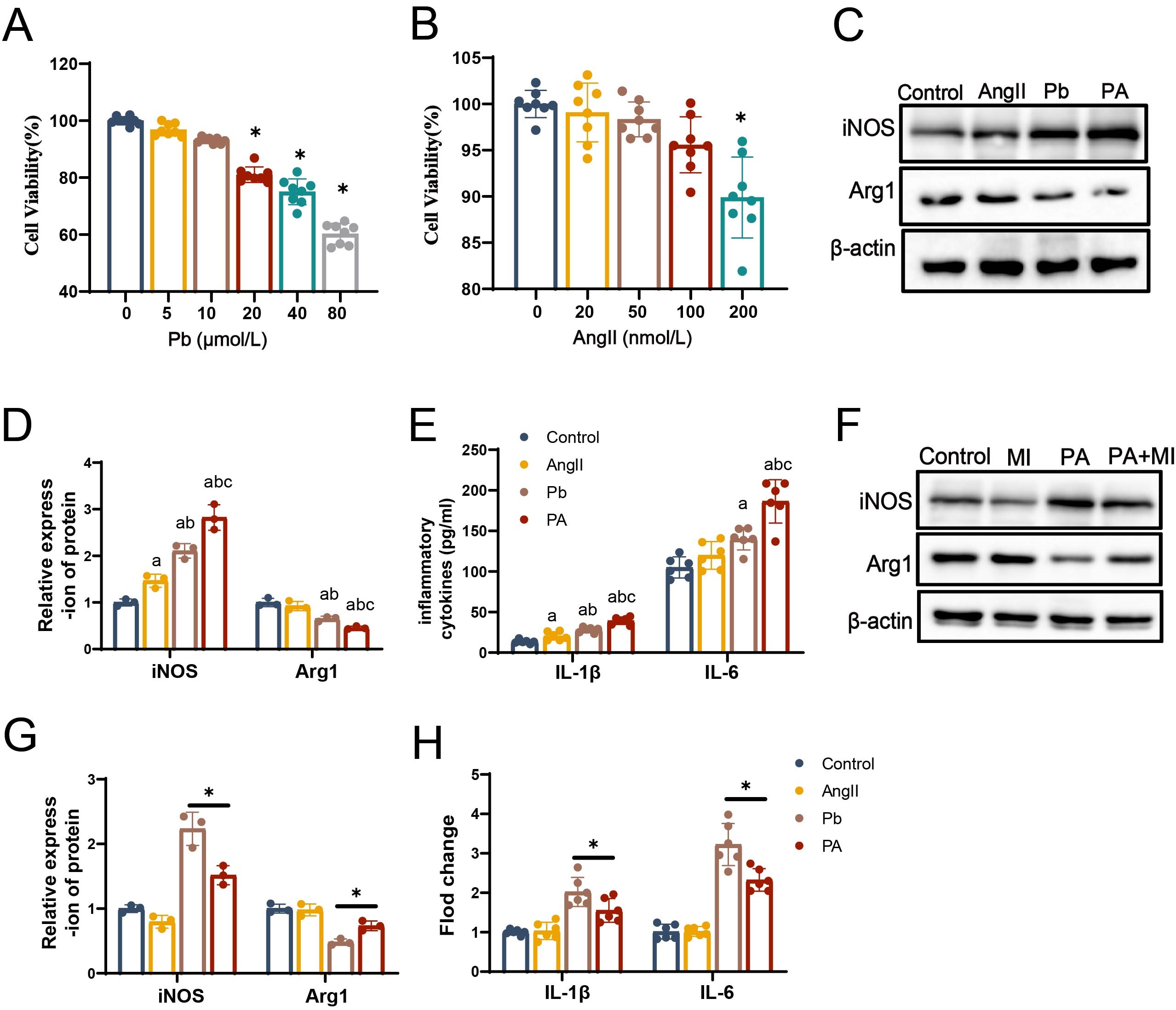

Supplement: Supplementary 1 — Figs. S1 to S5 Tables S1 to S4 Supplementary Materials 2—Blots [file research.1026.f1.zip › FIGS2.jpeg]

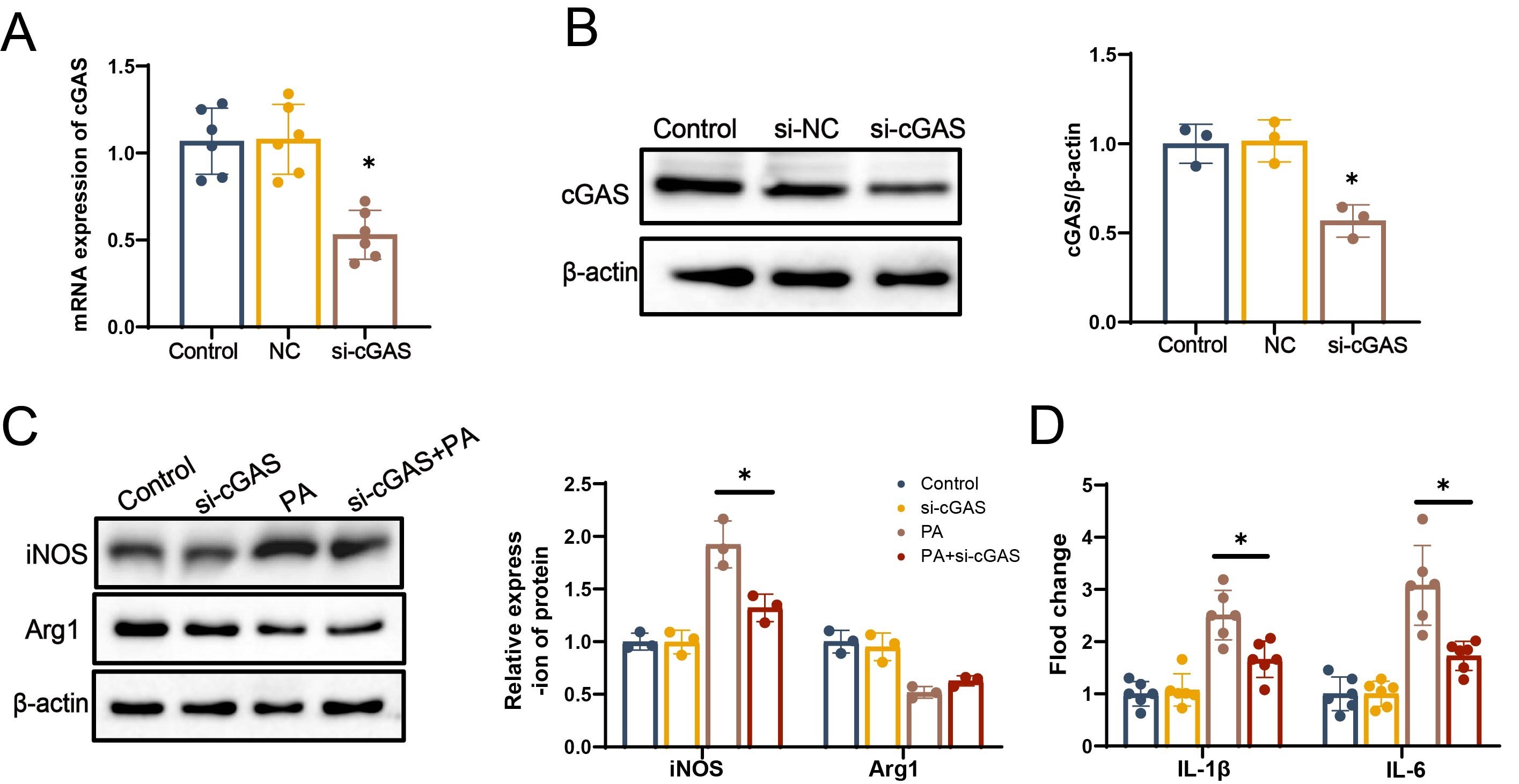

Supplement: Supplementary 1 — Figs. S1 to S5 Tables S1 to S4 Supplementary Materials 2—Blots [file research.1026.f1.zip › FIGS3.jpeg]

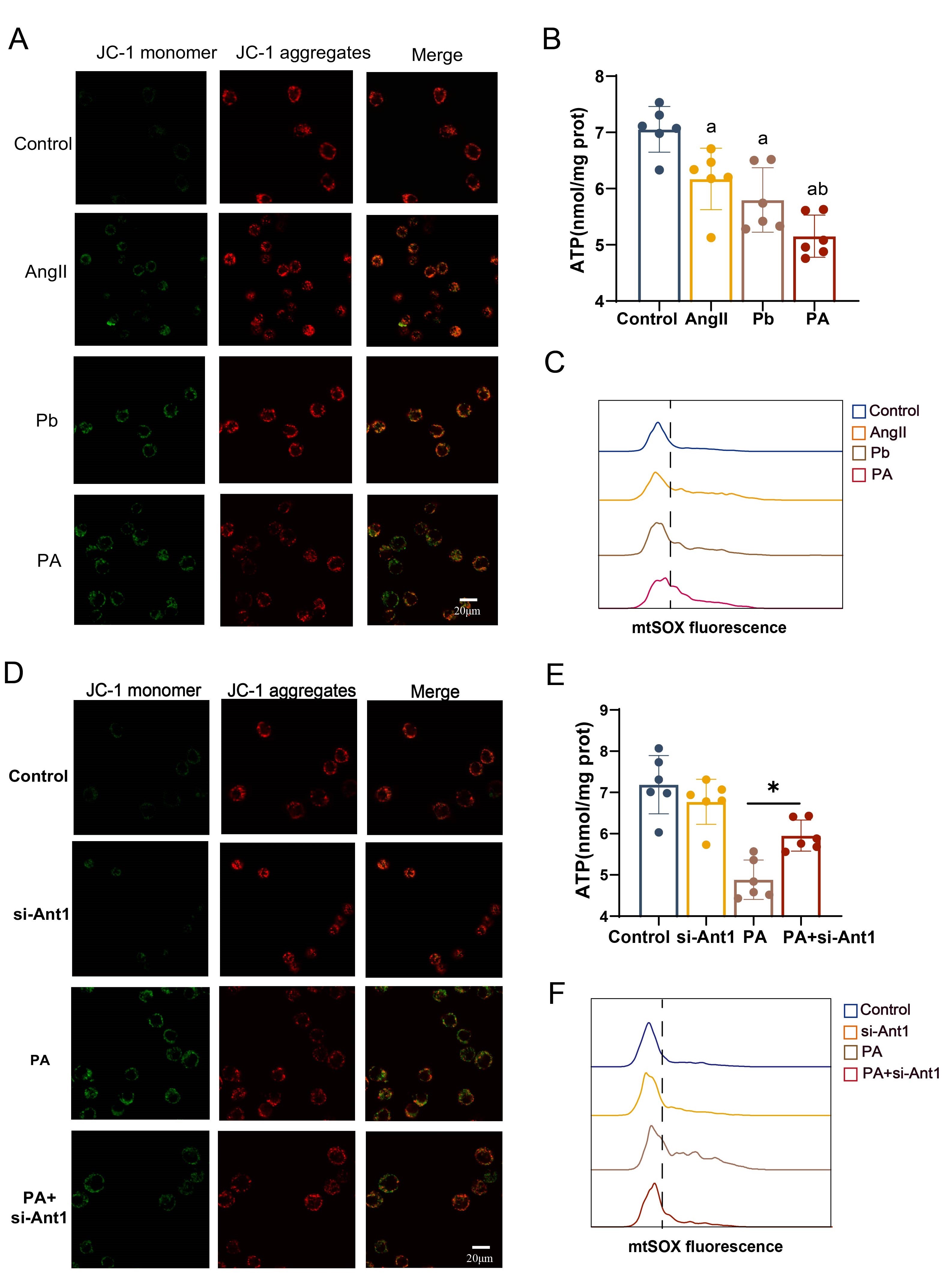

Supplement: Supplementary 1 — Figs. S1 to S5 Tables S1 to S4 Supplementary Materials 2—Blots [file research.1026.f1.zip › FIGS4.jpeg]

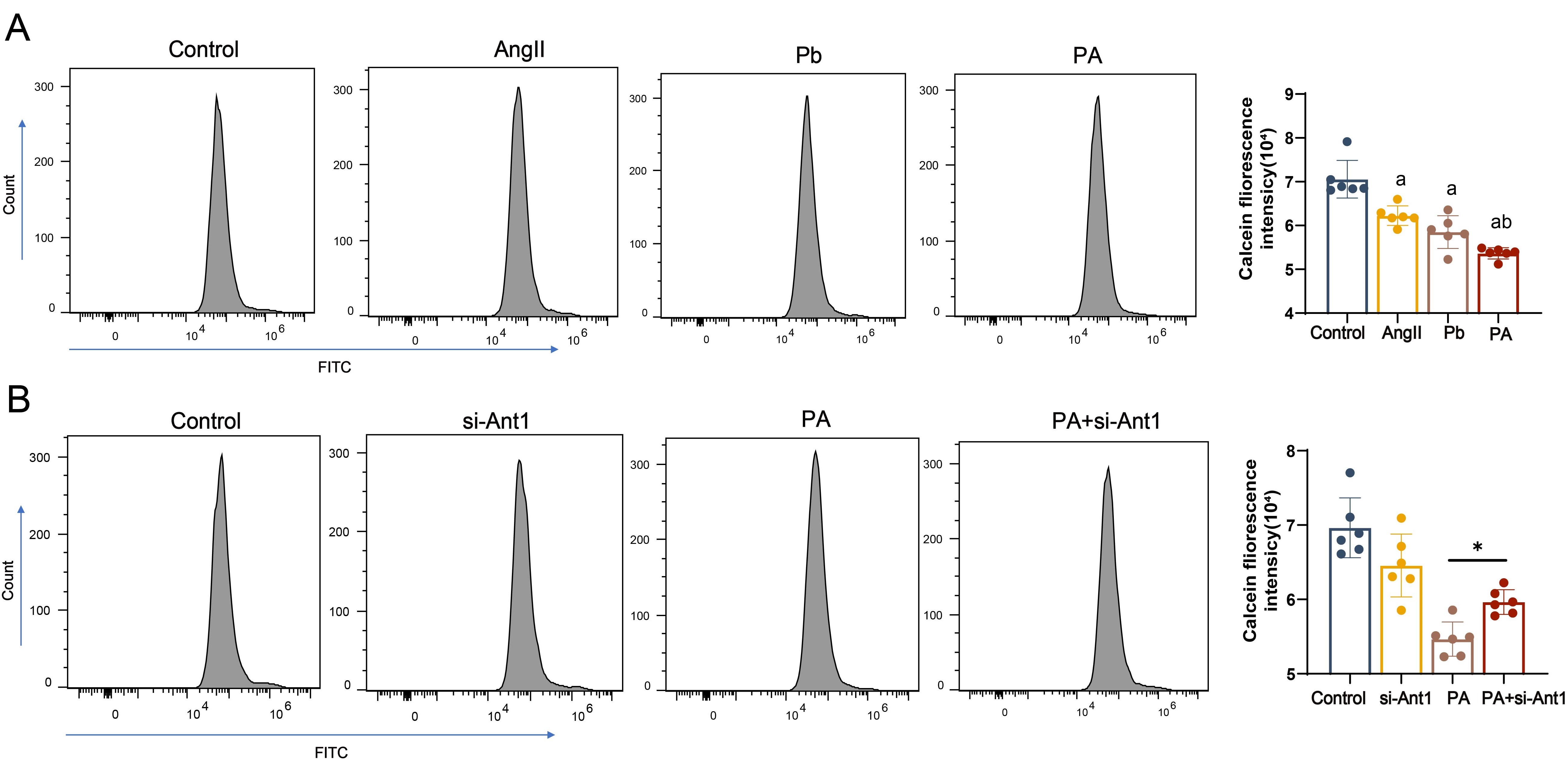

Supplement: Supplementary 1 — Figs. S1 to S5 Tables S1 to S4 Supplementary Materials 2—Blots [file research.1026.f1.zip › FIGS5.jpeg]

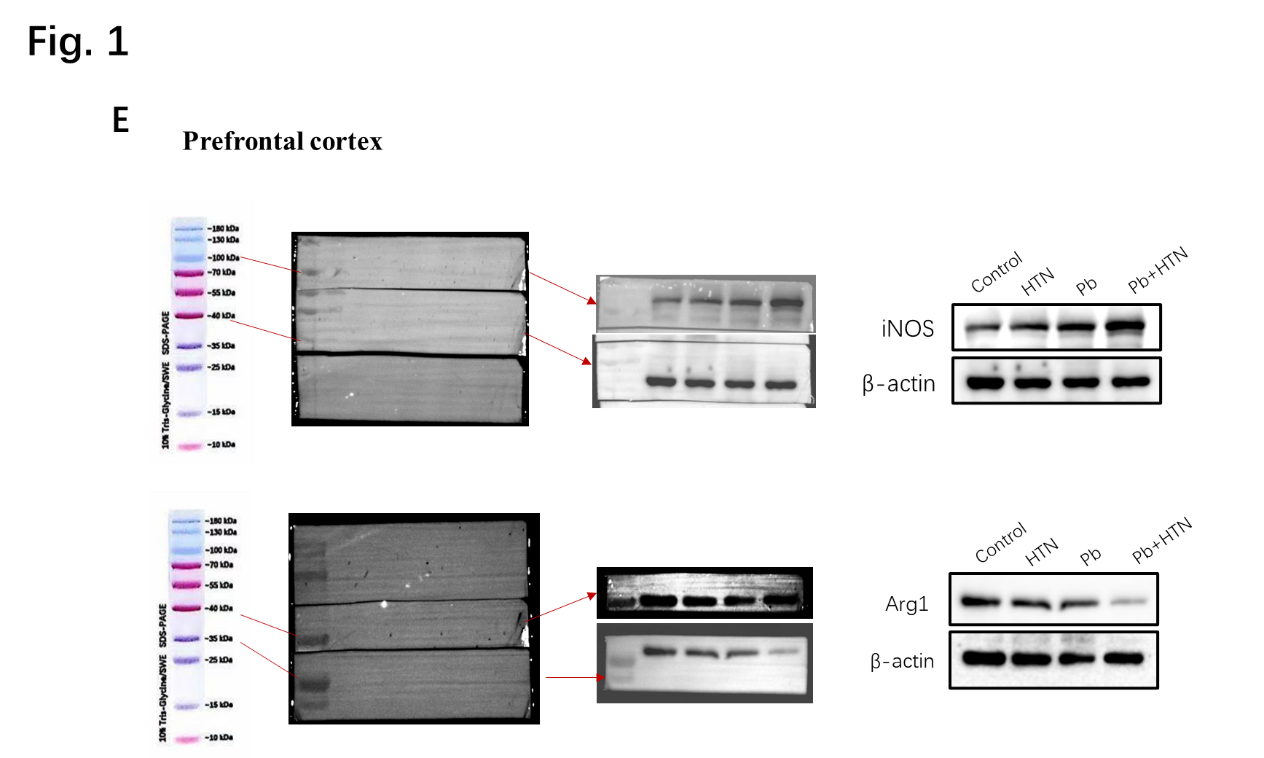


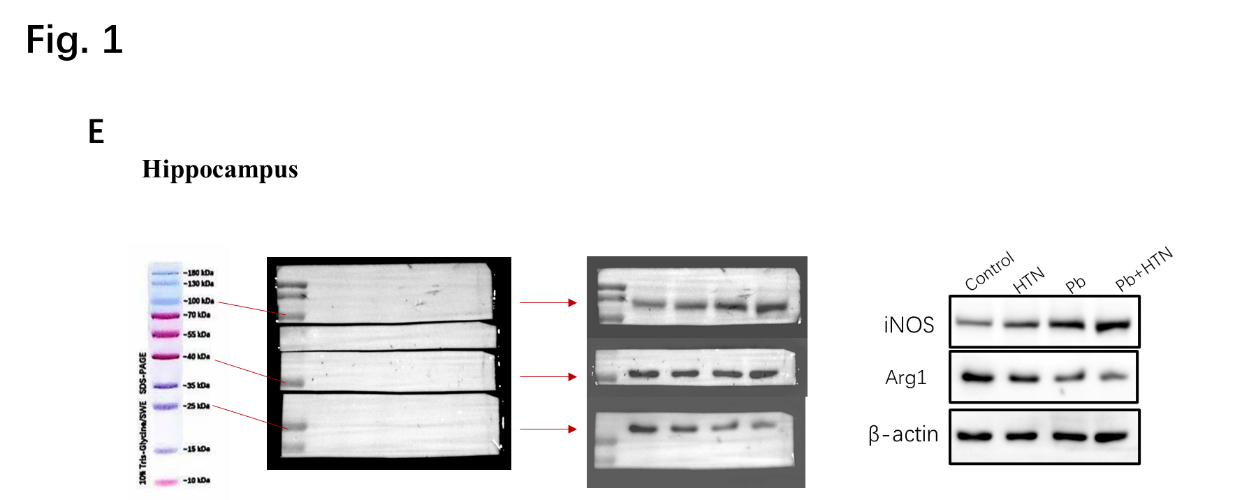


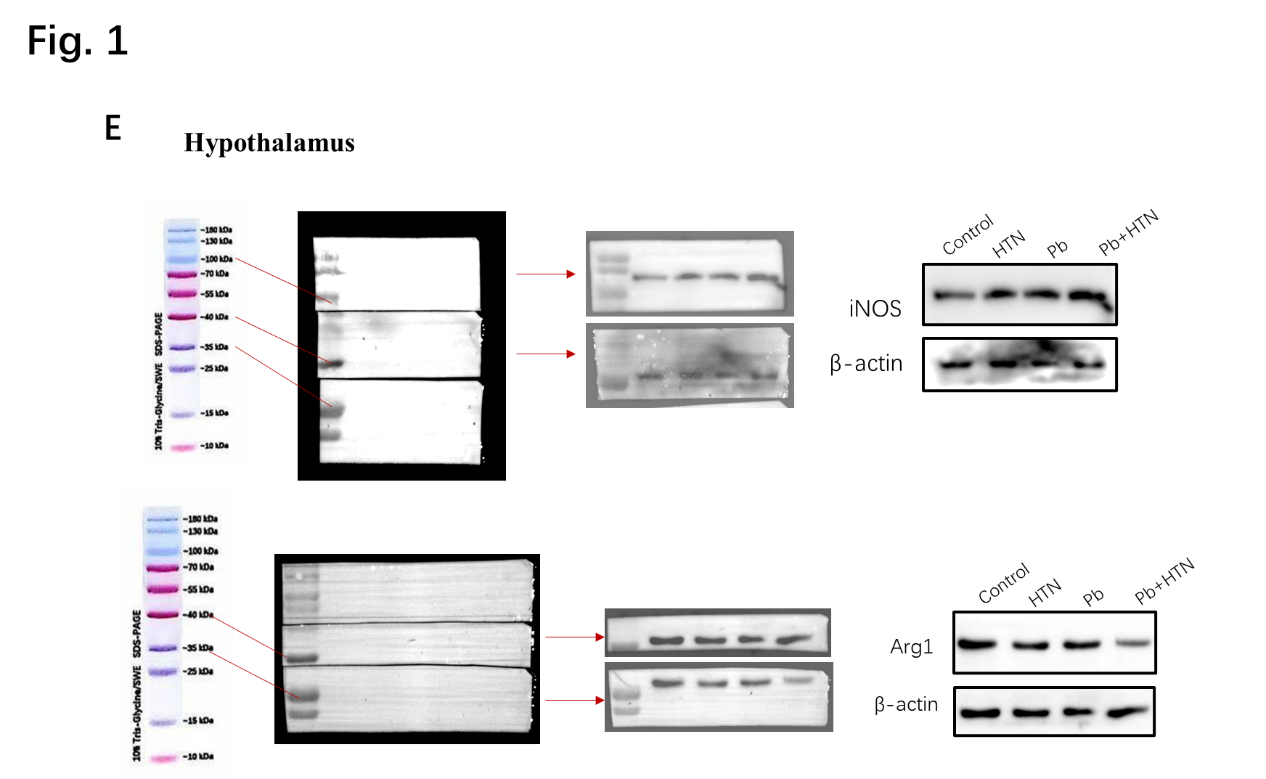


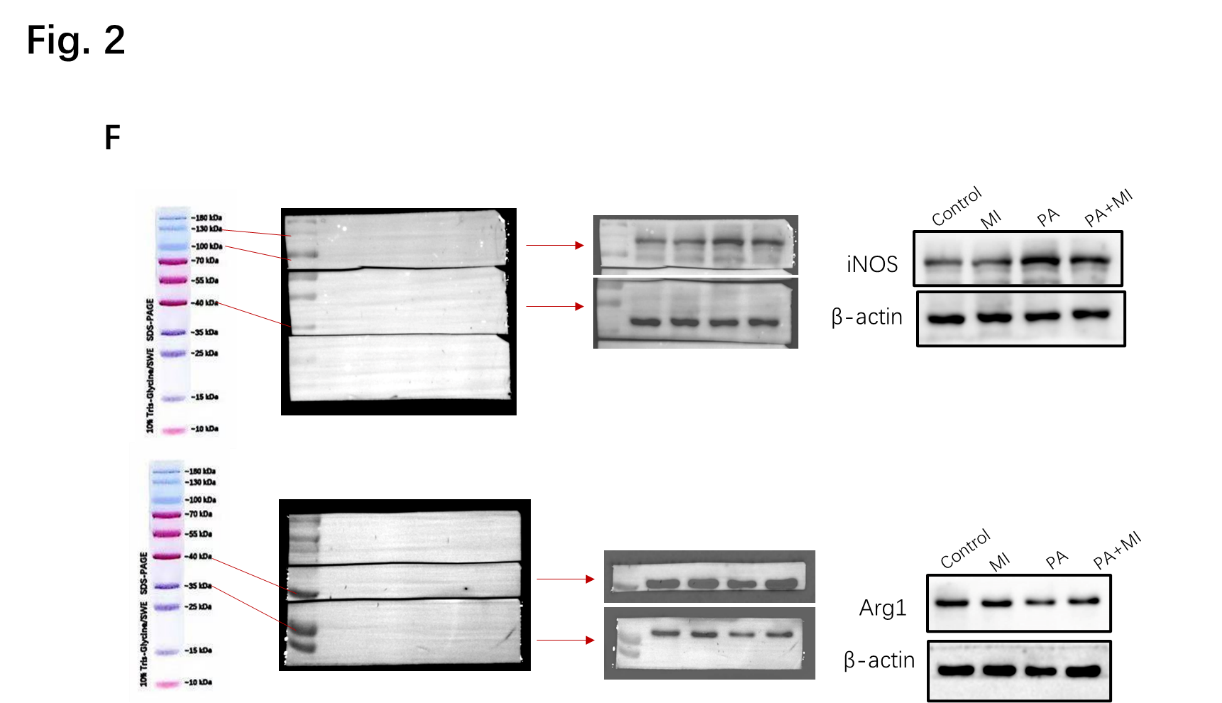


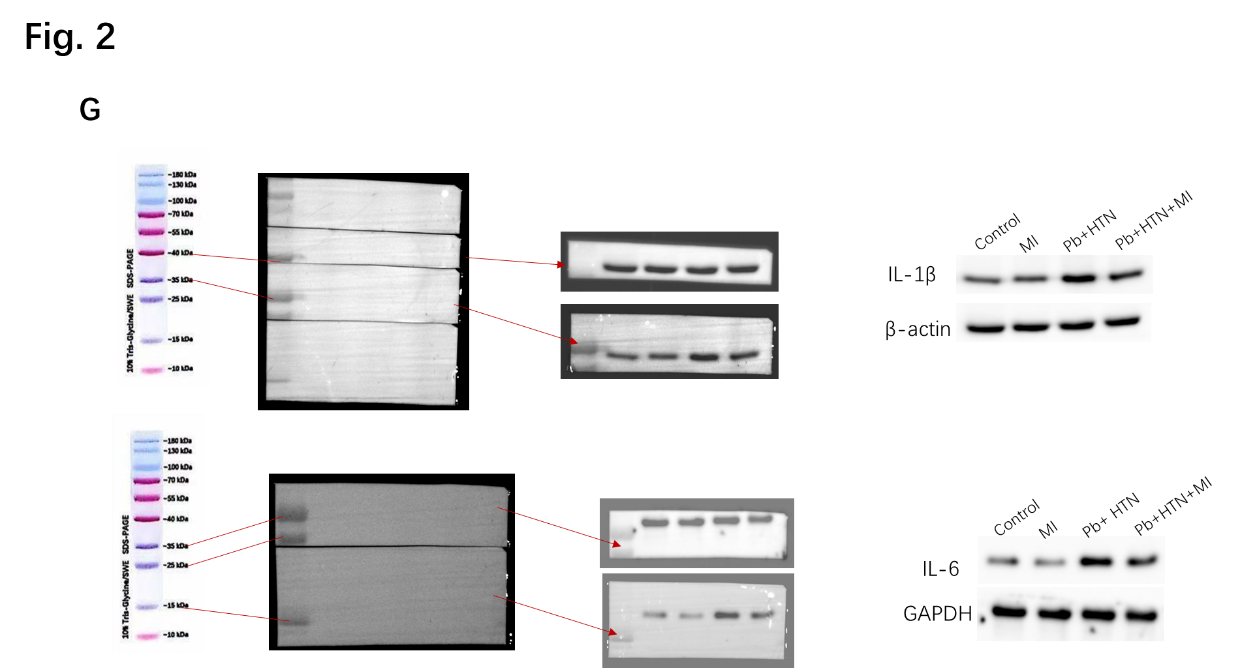


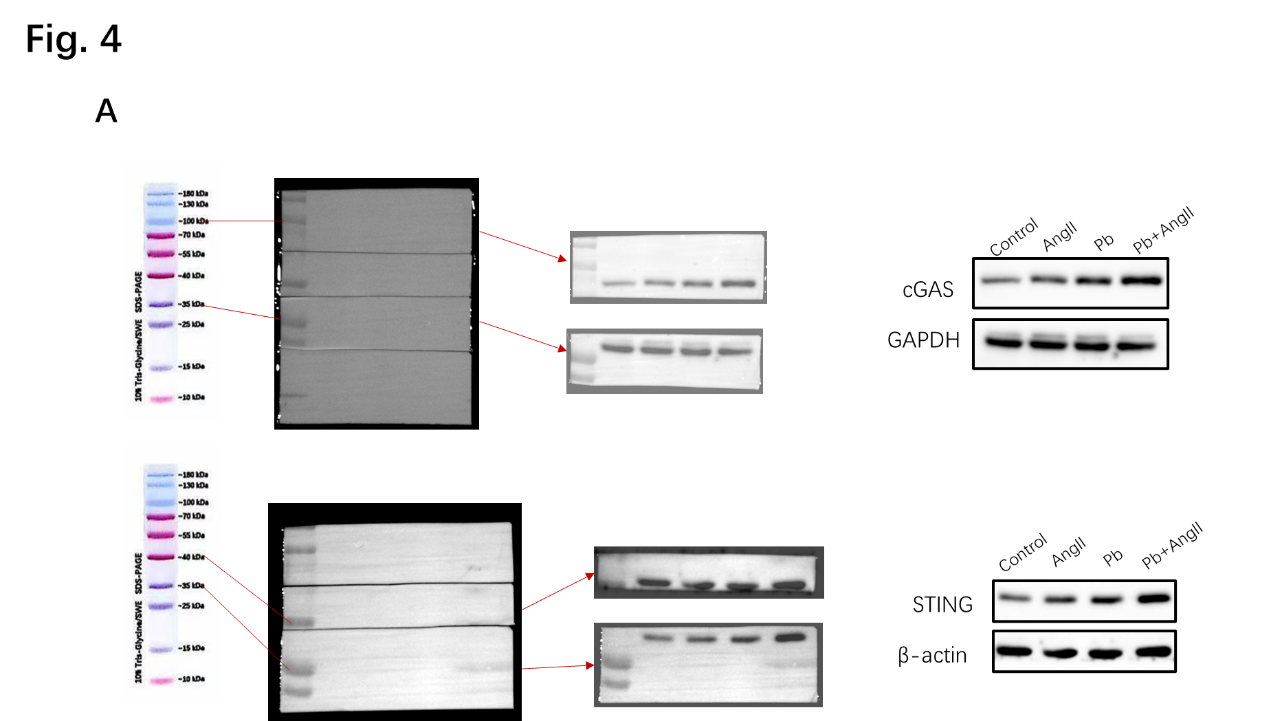


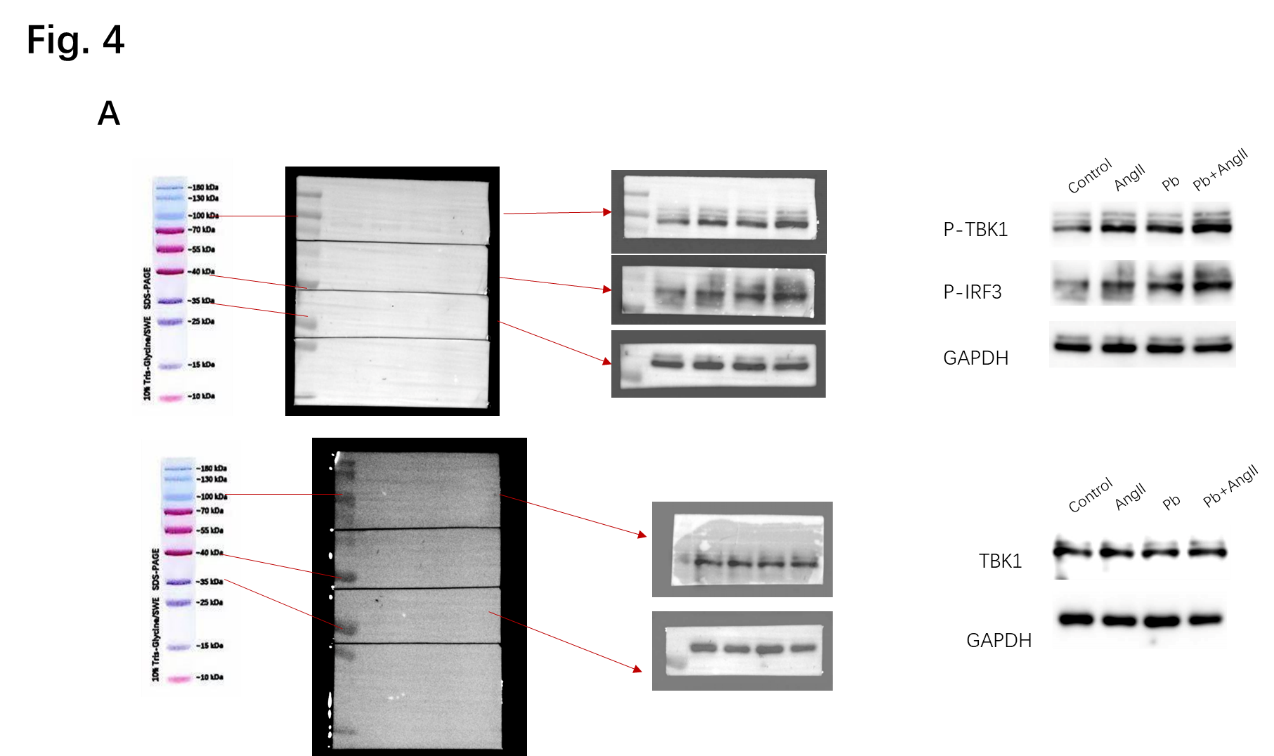


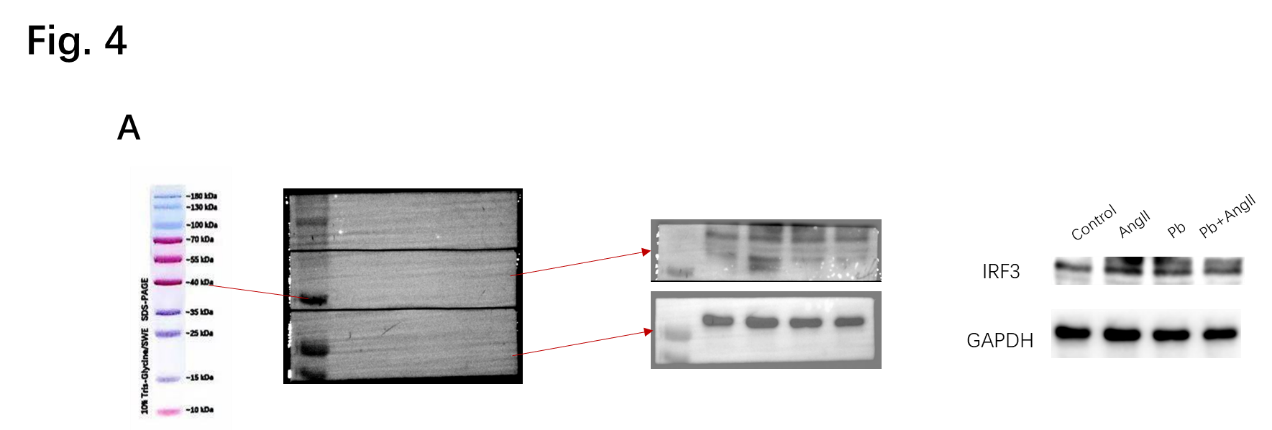


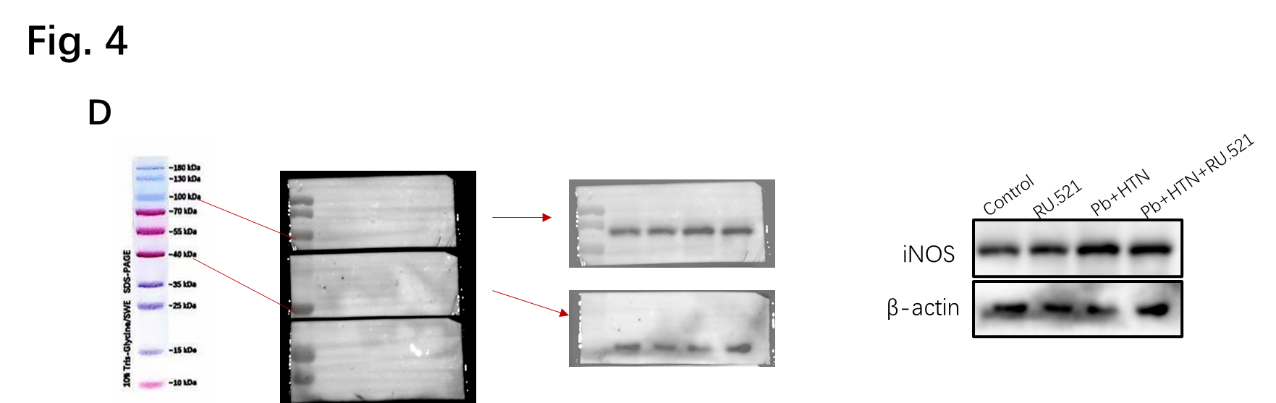


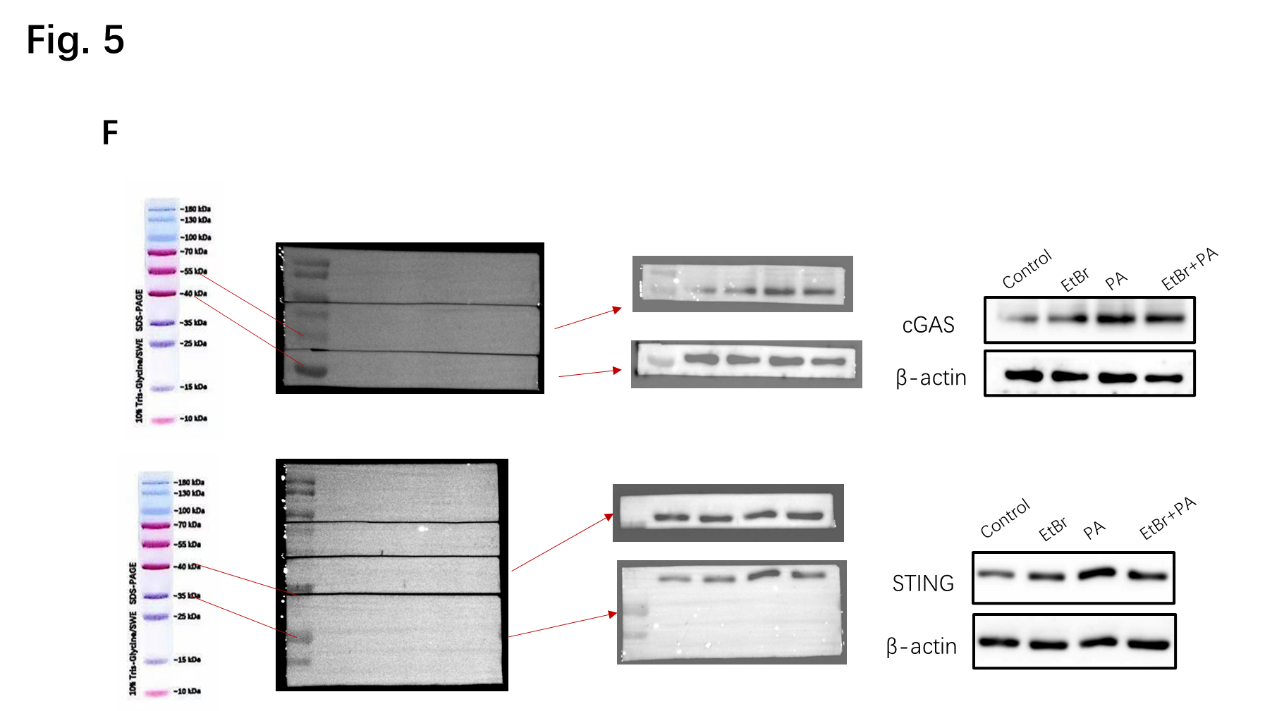


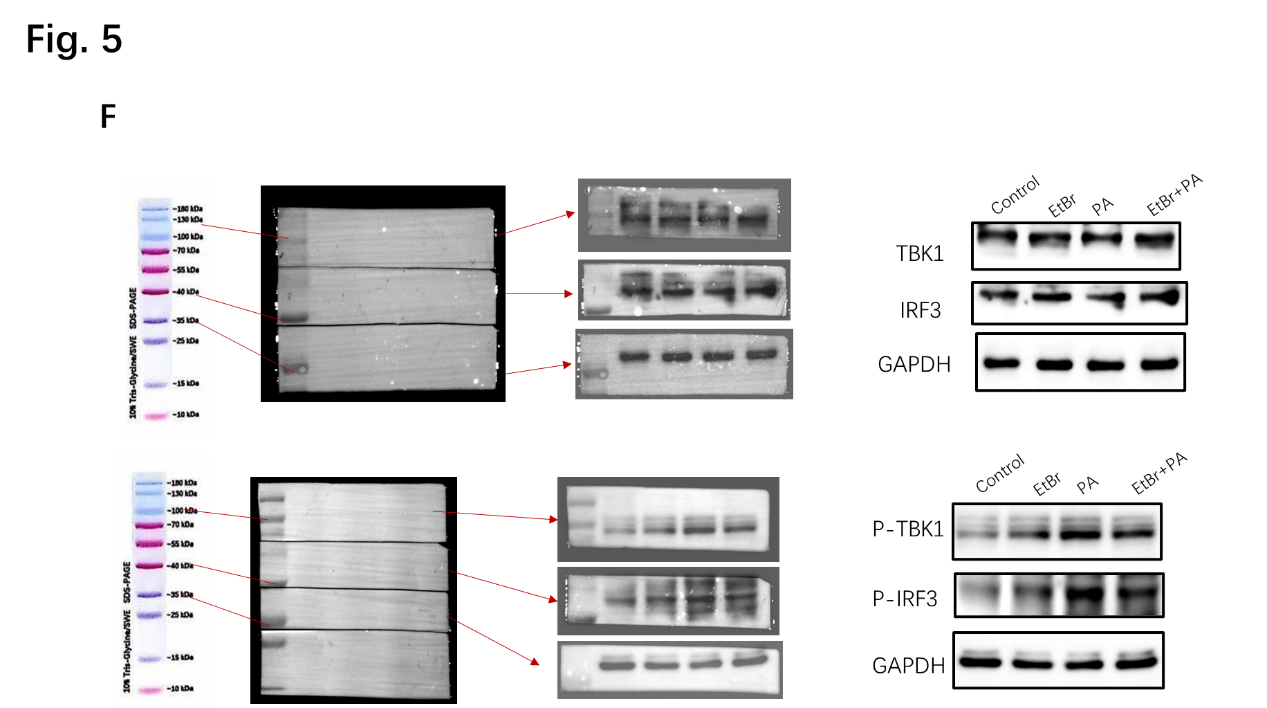


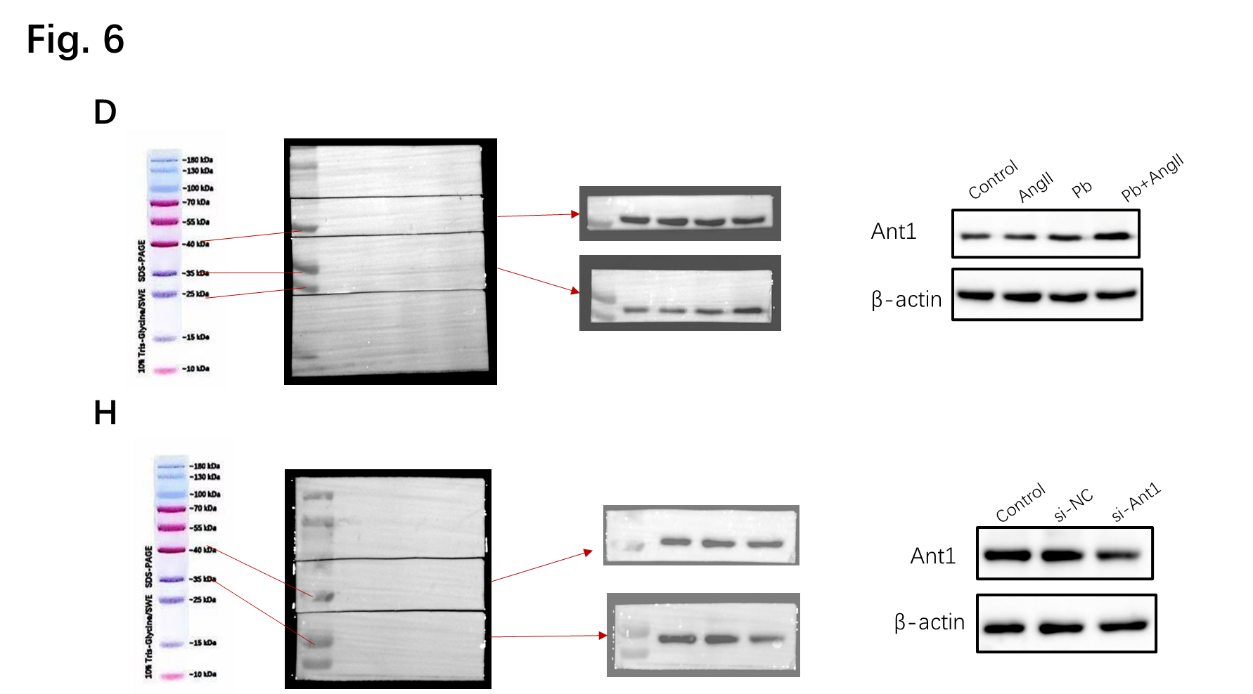


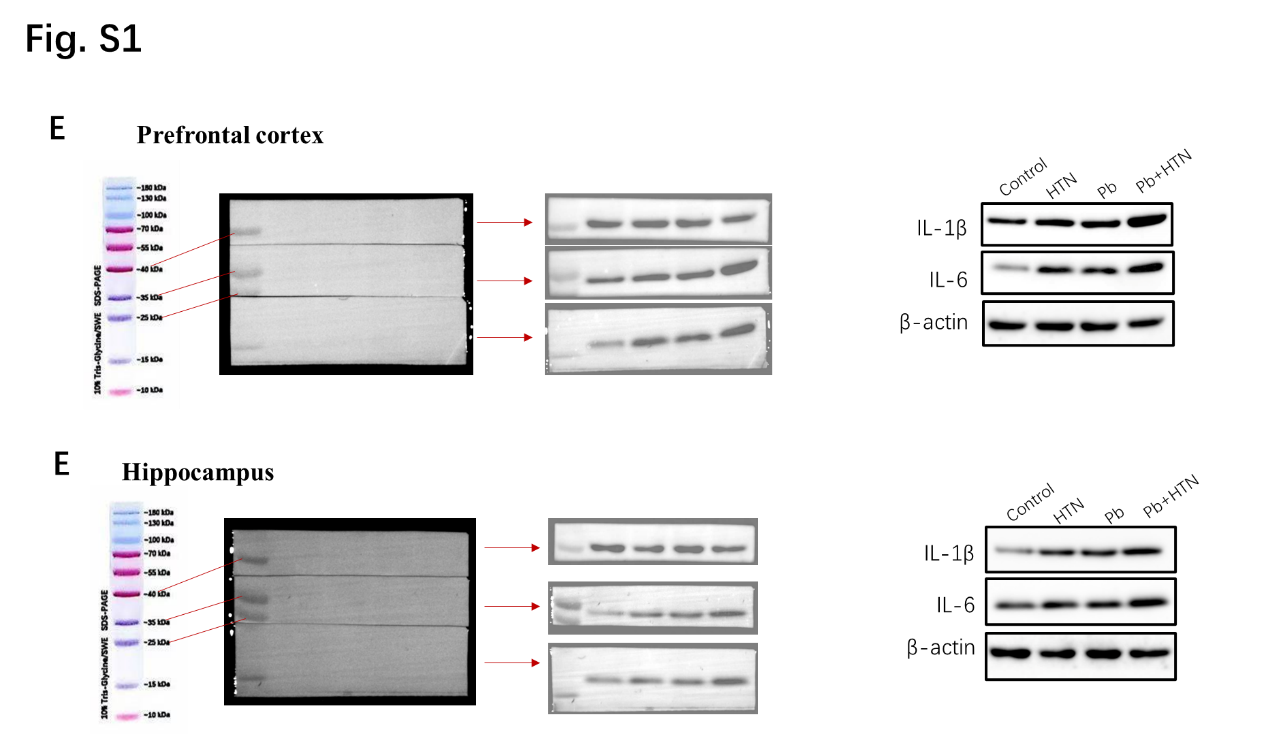


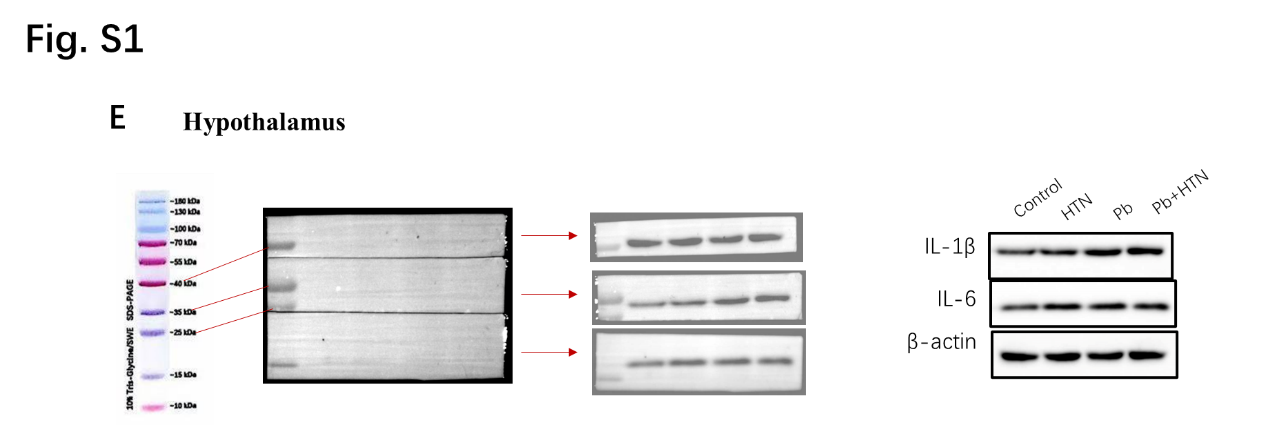


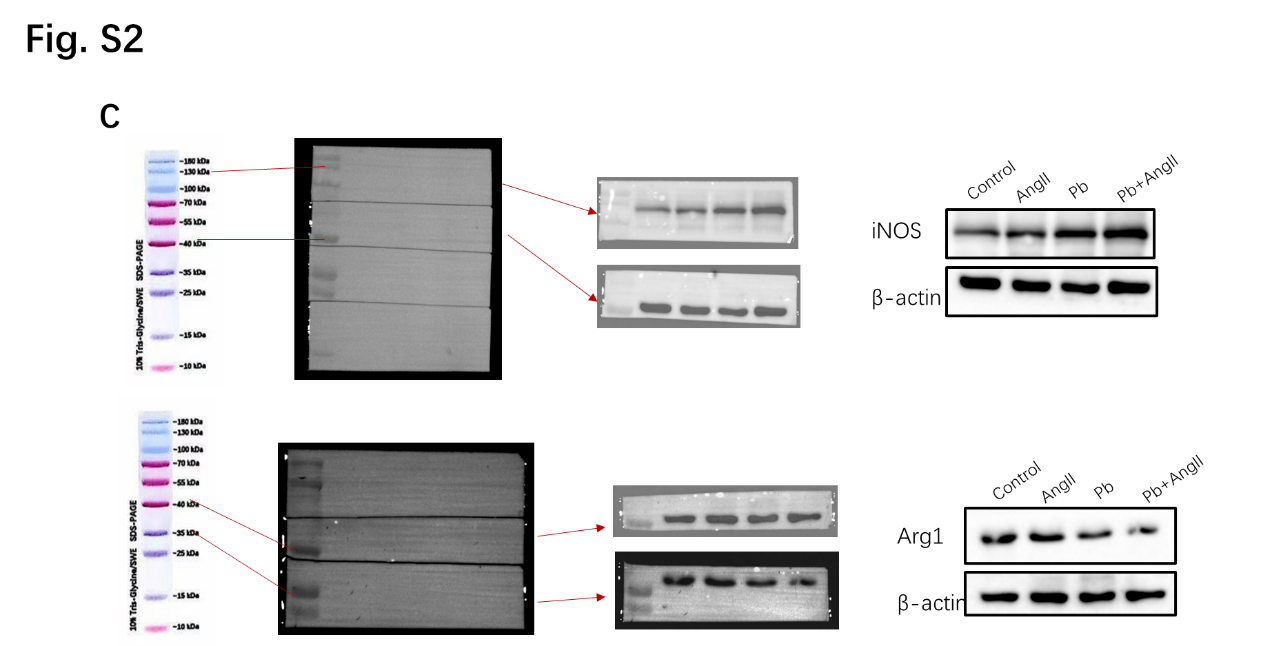


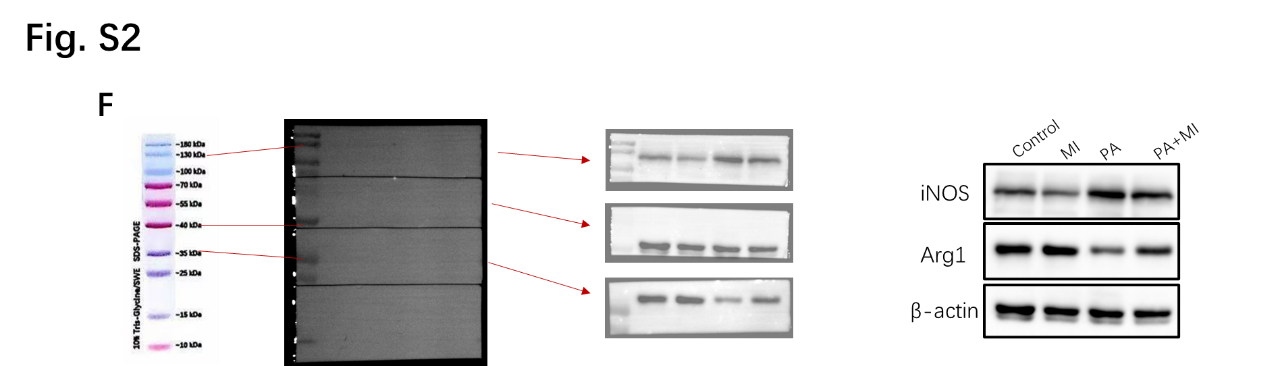


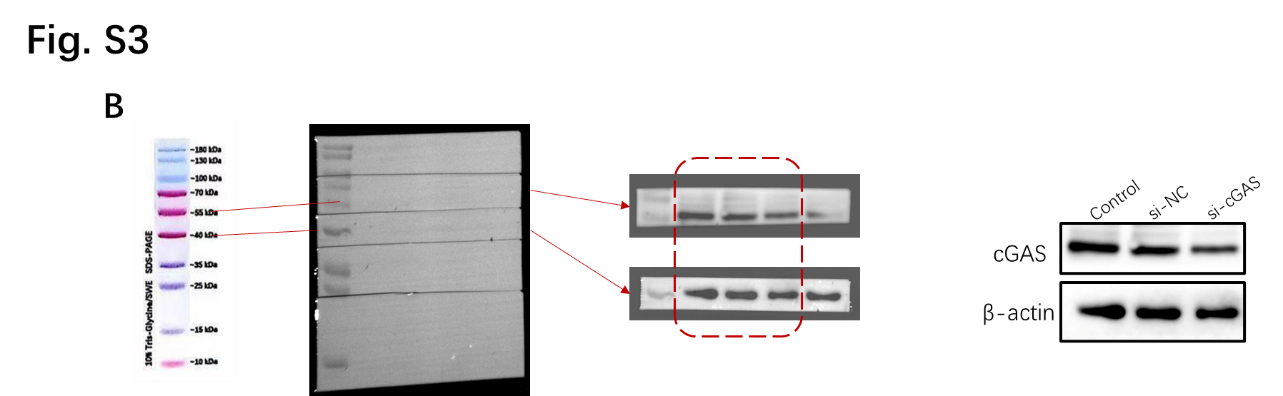


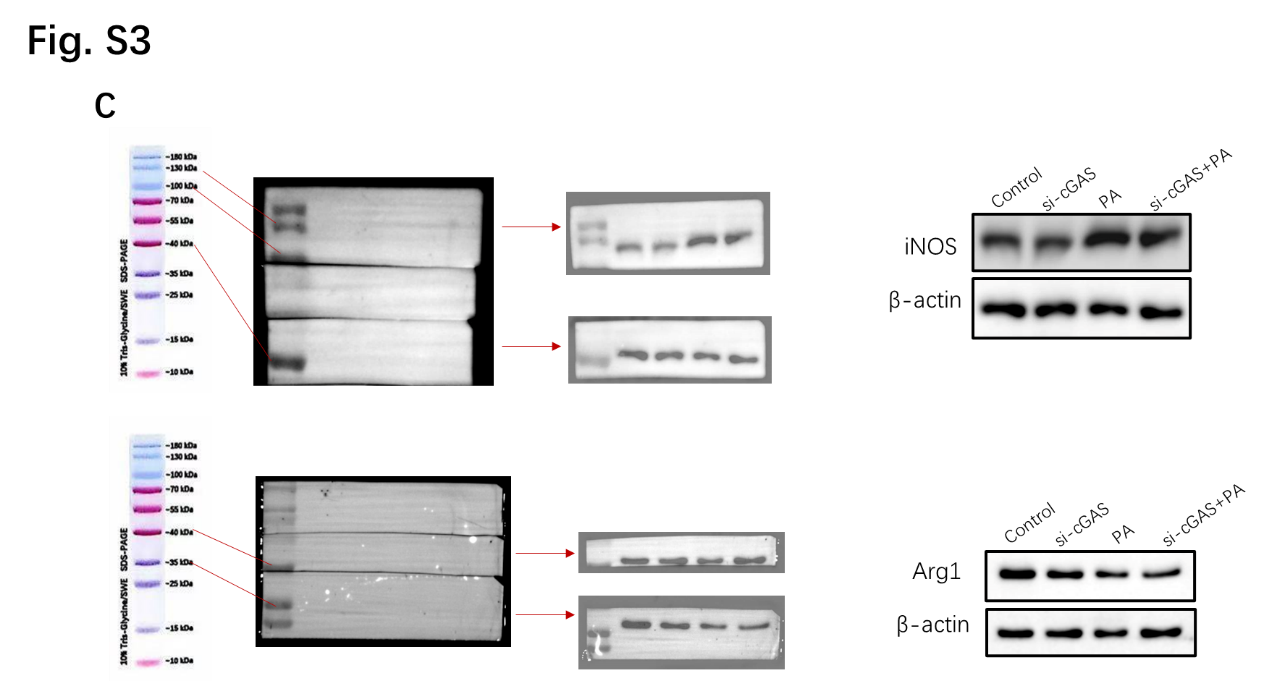

Supplement: Supplementary 1 — Figs. S1 to S5 Tables S1 to S4 Supplementary Materials 2—Blots [file research.1026.f1.zip › Supplementary materials 2-Blots.docx]
